# Trend of albumin nanoparticles in oncology: a bibliometric analysis of research progress and prospects

**DOI:** 10.3389/fphar.2024.1409163

**Published:** 2024-07-12

**Authors:** Ye Liu, Yi Li, Wei Shen, Min Li, Wen Wang, Xin Jin

**Affiliations:** ^1^ Department of Pharmacy, The Affiliated Suqian First People’s Hospital of Nanjing Medical University, Suqian, China; ^2^ Department of Pharmaceutics, State Key Laboratory of Natural Medicines, China Pharmaceutical University, Nanjing, Jiangsu, China; ^3^ Department of Rheumatology and Immunology, The Affiliated Suqian First People’s Hospital of Nanjing Medical University, Suqian, China

**Keywords:** albumin nanoparticles, anti-tumor, bibliometric research, hotspots, clinical applications

## Abstract

**Background:**

Delivery systems based on albumin nanoparticles (NPs) have recently garnered substantial interest in anti-tumor drug development. However, systematic bibliometric analyses in this field remain lacking. This study aimed to analyze the current research status, hotspots, and frontiers in the application of albumin NPs in the field of oncology from a bibliometric perspective.

**Methods:**

Using the Web of Science Core Collection (WOSCC) as the data source, retrieved articles were analyzed using software, such as VOSviewer 1.6.18 and CiteSpace 6.1.6, and the relevant visualization maps were plotted.

**Results:**

From 1 January 2000, to 15 April 2024, 2,262 institutions from 67 countries/regions published 1,624 articles related to the application of albumin NPs in the field of oncology. The USA was a leader in this field and held a formidable academic reputation. The most productive institution was the Chinese Academy of Sciences. The most productive author was Youn YS, whereas Kratz F was the most frequently co-cited author. The most productive journal was the *International Journal of Nanomedicine*, whereas the *Journal of Controlled Release* was the most co-cited journal. Future research hotspots and frontiers included “rapid and convenient synthesis methods predominated by self-assembly,” “surface modification,” “construction of multifunctional NPs for theranostics,” “research on natural active ingredients mainly based on phenolic compounds,” “combination therapy,” and “clinical applications.”

**Conclusion:**

Based on our bibliometric analysis and summary, we obtained an overview of the research on albumin NPs in the field of oncology, identified the most influential countries, institutions, authors, journals, and citations, and discussed the current research hotspots and frontiers in this field. Our study may serve as an important reference for future research in this field.

## 1 Introduction

Despite the considerable progress in the development of anti-tumor drugs, the mortality rate of cancer remains high owing to the low water solubility, poor bioavailability, major adverse reactions, and weak targeting of most anti-tumor drugs. Furthermore, tumor resistance and hyperprogression during drug treatment limit the effective use of these drugs. Therefore, developing more effective cancer treatment strategies is imperative. Currently, drug delivery systems are employed in clinical applications owing to their advantages in improving drug solubility, targeting, and half-life, as well as reducing systemic side effects.

Nano-delivery systems based on albumin have garnered widespread interest among researchers (Hong et al., 2020; Martínez-López et al., 2020). Albumin, the most abundant protein in plasma, accounting for approximately 50% of total plasma protein, is endogenous and multifunctional. In the physiological environment, it acts as a natural transport carrier in the blood and can reversibly bind to various endogenous and exogenous molecules, including long-chain fatty acids, bilirubin, iron, calcium, taxanes, sulfonamides, and other drugs, which are then transported to various tissues (Kouchakzadeh et al., 2015). Since Peters first described the structure, molecular genetics, and *in vivo* metabolism of albumin in 1985, subsequent research has revealed its numerous advantages, including biodegradability, good biocompatibility, non-immunogenicity, and a long half-life (Peters, 1985). Consequently, albumin-based drug delivery systems have gained attention in the field of oncology.

Albumin and albumin-drug complexes mainly enhance their accumulation in tumor tissues through the following pathways:

1) Dysopsonins. Opsonins, proteins found in serum, can enhance phagocytosis by phagocytes. They can be adsorbed to the surface of nanoparticles (NPs), making them more vulnerable to phagocytosis and ultimately shortening their half-life. Dysopsonins, however, are a class of proteins that can extend the half-life of NPs within the body. Dysopsonins exhibit a relatively low affinity for the cell surface. They inhibit the further adsorption of opsonins or block the adsorption sites of opsonins, reducing the immunogenicity of NPs and exhibiting a stealth effect. Encapsulating drugs within albumin, which is a type of dysopsonin, reduces the adsorption of opsonins and inhibits phagocytosis by phagocytes, extending the half-life of drugs *in vivo* (Wang and Zhang, 2022).
2) Enhanced permeability and retention (EPR) effect. Tumor tissue blood vessels have large gaps, poor structural integrity, and lack lymphatic vessels, allowing albumin to accumulate in tumor tissues without being removed by the return of lymph fluid. Therefore, albumin and albumin–drug complexes can passively target tumor tissues based on the EPR effect (Bertrand et al., 2014).
3) Macropinocytosis. Tumor cells actively take up extracellular albumin through macropinocytosis to support the supply of amino acids and energy required for their growth (Commisso et al., 2013).
4) Albumin receptors. Tumor cells can also transport albumin to certain tumor tissues through albumin-mediated transendothelial cell transport (Hoogenboezem and Duvall, 2018).

The above pathways facilitate the accumulation and uptake of albumin and albumin–drug complexes at tumor sites.

Albumin binds to drugs through four main methods: human serum albumin (HSA)–drug NPs, HSA–drug conjugates, HSA-binding prodrugs, and HSA-based recombinant fusion proteins (Tao et al., 2021). The latter three methods have limited applicability and low drug-loading efficiency, as their drug-loading capacities are significantly constrained by the physiochemical properties of the drug. However, encapsulating drugs in albumin to form albumin NPs can circumvent these drawbacks, achieving a drug-loading method with wide applicability. This method is suitable for various therapeutic molecules that cannot be noncovalently or covalently linked with albumin or undergo gene fusion. It also allows for the simultaneous encapsulation of multiple drugs and imaging agents to implement combination therapy. In addition, albumin NPs can be modified to increase their accumulation at specific sites.

Currently, two anti-tumor drugs—Abraxane^®^ (Bristol-Myers Squibb, Princeton, NJ, USA) and Fyarro^®^ (AADI Bioscience, Pacific Palisades, CA, USA)—are delivered using albumin NPs. On 7 January 2005, the US Food and Drug Administration (FDA) approved Abraxane^®^, a type of albumin-bound paclitaxel NPs, to treat metastatic breast cancer after failed combination chemotherapy. Abraxane^®^ was later approved in 2012 and 2013 for the first-line treatment of non-small cell lung cancer and metastatic adenocarcinoma of the pancreas, respectively. Abraxane^®^ was the first successful case of albumin nanoparticle (NP) drug delivery technology, leading to increased clinical research on other albumin NP-based drug delivery formulations in the field of oncology (Meng et al., 2023). On 23 November 2021, the FDA approved Fyarro^®^, an albumin-bound NP formulation of sirolimus, to treat locally advanced unresectable or metastatic malignant perivascular epithelioid cell tumor (PEComa). This is the first FDA-approved drug for treating advanced malignant PEComa in adults and the second anti-tumor product developed and marketed based on albumin technology.

The growing number of studies on albumin NPs as carriers of anti-tumor drugs has led to the development of various methods for drug–albumin conjugation and discovery of many new drugs that can be conjugated with albumin to form NPs, enhancing their anti-tumor activity and targeting. This has laid a solid theoretical foundation for basic research and the rational development and utilization of albumin NPs as targeted carriers of anti-tumor drugs. Several reviews exist on albumin-based nanodrugs, covering aspects such as preparation techniques, surface modifications, construction of multifunctional NPs, and therapeutic applications. These reviews summarize albumin-based nanomedicine and have promoted the rapid technological development of this field. However, studies presenting intuitive analysis and summary of the development trends, publishing institutions, research authors, research hotspots, and frontiers in the oncological applications of albumin NPs remain lacking.

**FIGURE 1:**
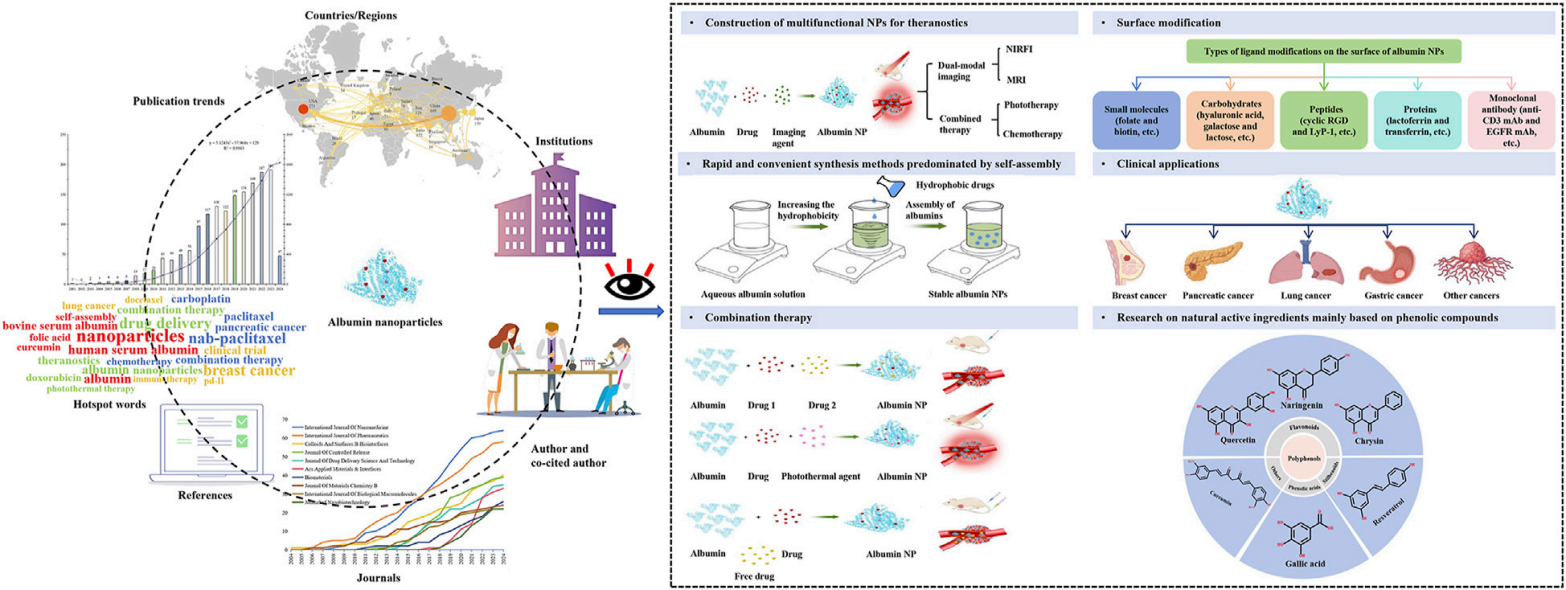
A bibliometric analysis of the research progress and prospects of albumin nanoparticles in oncology.

The information age has facilitated the rapid development of bibliometrics, a field that focuses on mining, analyzing, and summarizing the course and structure of knowledge development over time and space. Recently, the fastest-growing area of bibliometric research has been the quantitative and qualitative analysis of literature related to a specific research direction, to reveal research trends, hotspot distribution, and status (Pei et al., 2022; Gu et al., 2023; Ling et al., 2023; Zhiguo et al., 2023). This study aimed to conduct bibliometric research on the use of albumin NPs in oncology within English-language literature using software, including CiteSpace (v6.1.6; https://citespace.podia.com/) and VOSviewer (v1.6.18; https://www.vosviewer.com/). The literature retrieved was used to create knowledge maps, such as co-authorship networks, institutional collaboration networks, co-citations, co-words, and keyword clustering. These maps were then analyzed visually to explore current research hotspots and frontiers (Figure 1). This research may help experts and novices intuitively determine the breadth of their fields, find new topics of interest, gain intimate knowledge of clinical application, and formulate future research plans, thereby providing great convenience for researchers.

## 2 Materials and methods

### 2.1 Data source

All studies were retrieved and downloaded from the Web of Science Core Collection (WOSCC) database. The WOSCC is one of the most authoritative citation databases in the scientific community. It contains a curated collection of over 21,000 globally recognized, high-impact academic journals, over 200,000 conference proceedings, and the bibliographic abstracts of over 120,000 scientific and technical books, with topics spanning the natural sciences, engineering technology, biomedicine, social sciences, arts and humanities, and other fields. Articles in the WOSCC represent the position of medical science, and numerous existing articles on bibliometric analyses and visualization of scientific literature have used the WOSCC as their data source (Pei et al., 2022; Xu et al., 2023; Cao et al., 2024). Therefore, we selected the WOSCC as the data source for this study. The search queries were as follows: TS = “nanoparticle albumin” OR “albumin-based nanoparticle” OR “albumin-based nanoparticles” OR “albumin based nanoparticle” OR “albumin based nanoparticles” OR “(BSA) nanoparticle” OR “(BSA) nanoparticles” OR “BSA NP” OR “BSA NPs” OR “BSA nanoparticle” OR “BSA nanoparticles” OR “albumin NPs” OR “albumin nanoparticles” OR “albumin nanoparticle” OR “ALB nanoparticle” OR “ALB nanoparticles” OR “HSA nanoparticle” OR “HSA nanoparticles” OR “(HSA) nanoparticle” OR “(HSA) nanoparticles” OR “HSA NP” OR “HSA NPs” or “albumin-bound nanoparticle” OR “albumin-bound nanoparticles” OR “albumin-stabilized nanoparticle” OR “albumin-stabilized nanoparticles” OR “albumin-stabilized nap” OR “albumin-stabilized naps” AND TS = “tumor” OR “neoplasm” OR “tumors” OR “neoplasia” OR “neoplasias” OR “cancer” OR “cancers” OR “anticancer” OR “anti-cancer” OR “antitumor” OR “anti-tumor”. The search date range was between 1 January 2000, and 15 April 2024.

### 2.2 Screening of the literature

Following the initial data search, two researchers independently reviewed the title, abstract, keywords, author information, and main text of the articles to ensure their relevance to the topic of our study. Only original research articles and review papers were included in this study, and letters, briefings, and book reviews were excluded. In cases of disagreement among the selections, other group members were consulted. Notably, 1,624 articles related to the topic were selected (Supplementary Figure S1).

### 2.3 Methods of visualization

Bibliometric methods were used to perform visualization analyses on the authors, institutions, countries, journals, co-citations, and co-words of the retrieved literature using the VOSviewer 1.6.18, CiteSpace 6.1.6, Pajek (http://mrvar.fdv.uni-lj.si/pajek/), and Scimago Graphica (https://www.graphica.app/) software. The resulting visualization maps were analyzed to determine the status, hotspots, and trends of research.

## 3 Results

### 3.1 Publication trends

Statistical analysis was performed on the retrieved articles to reflect changes in the number of articles published on albumin NPs in the field of oncology (Supplementary Figure S2). From 1 January 2000, to 15 April 2024, 1,624 articles were published on this topic (as no articles were published in 2000, those data were not included in the figure). The average number of articles published per year was 68.57 (2001–2023), with an average annual growth rate of 26.96 (2001–2023). The overall development trends can be roughly divided into three phases, as described in the following paragraphs.

During Phase 1 (2001–2007), fewer than three articles were published per year. Notably, on 7 January 2005, the FDA approved Abraxane^®^, the first drug synthesized with albumin NP drug delivery technology, for second-line treatment of metastatic breast cancer (Shi et al., 2017).

During Phase 2 (2008–2014), Abraxane^®^ achieved great success and contributed to the growing body of research on albumin NPs as anti-tumor drug carriers. The number of articles published on this topic increased compared with that of the previous phase, although the overall number of publications remained relatively low, with an average of 34.57 articles per year, which may primarily be attributed to the fact that albumin nanodrugs fall under the category of nano-preparations, which presents technical barriers and research challenges.

During Phase 3 (2015–2023), there was a trend of high-level growth and fluctuations, with an average of 146.11 articles published per year, a significant increase from the previous phase. Furthermore, a new albumin NP—sirolimus albumin-bound NP (Fyarro^®^) was introduced on 23 November 2021, for the treatment of locally advanced unresectable or metastatic malignant PEComa. The launch of this drug succeeded and provided a boost to the field of albumin NP drug delivery technology.

An exponential function was created to represent the annual publication trend: y = 5.1243x^2^ − 57.964x + 129 (*R* ^2^ = 0.9943, where x is the year and y is the number of publications per year). This function showed good agreement with the growth of annual publications. Based on this curve, we predicted that the number of articles published each year will steadily increase, indicating a growing interest among researchers in albumin NPs as anti-tumor drug carriers.

### 3.2 Distribution of countries/regions

Articles related to the application of albumin NPs in the field of oncology were published in 67 countries. China was the most productive country, with 649 articles, followed by the USA with 273 articles. These two countries accounted for 56.77% of the articles published in this field. In Table 1, China ranked first in terms of H-index of 64, followed by the USA of 60, India of 35, Germany of 34, and South Korea of 33. In terms of the average number of citations per article, the top five countries were Germany with 247.27, Australia with 180.44, Spain with 101.93, South Korea with 90.68, and the USA with 75.62. The USA had a high number of citations, H-index, and average citations per article, indicating its positive impact and importance in this field. As shown in Table 1, China had the highest number of publications and the second highest number of total citations among the top 10 countries. However, it ranked only eighth in terms of the average number of citations per article. In contrast, Germany had a much lower number of published articles, only 9.71% of China’s, but had the highest average number of citations per article, seven times that of China, and ranked first among the top 10 countries. These findings suggest that although China has published numerous articles, the quality of those articles was inadequate. In contrast, Germany published high-quality articles that served as valuable references.

**TABLE 1:** The 10 most productive countries related to research on albumin NPs in oncology.

Rank	Country	Counts	Citations	TLS	H-index	Average citation per paper	SCP	MCP	MCP_Ratio
1	China	649	20,633	122	64	31.79	564	59	0.095
2	United States of America	273	20,645	201	60	75.62	146	49	0.251
3	India	152	3,961	63	35	26.06	110	20	0.154
4	Japan	139	8,376	44	24	60.26	124	7	0.053
5	Iran	119	3,294	51	31	27.68	87	19	0.179
6	South Korea	93	8,433	57	33	90.68	70	12	0.146
7	Germany	63	15,578	86	34	247.27	33	14	0.298
8	Italy	51	3,419	47	21	67.04	31	11	0.262
9	Spain	40	4,077	59	20	101.93	19	11	0.367
10	Australia	39	7,037	70	21	180.44	14	5	0.263

The single country publications (SCP) value refers to the number of articles co-written by authors of the same nationality, whereas multiple country publications (MCP) value refers to the number of articles co-written by authors from different countries. The MCP ratio represents the level of global collaboration. China and the USA exhibited the highest MCP values, indicating a high level of collaboration among the top 10 countries with the most publications. However, the MCP ratios of all countries were relatively low, suggesting limited collaboration among different countries.

Visualization analysis was conducted for the publication regions (Supplementary Figure S3). The analysis revealed that the USA had the highest total intensity of cooperation. Further analysis indicated that the USA and China had the closest cooperation. Furthermore, according to the citation burst map (Supplementary Figure S4), Saudi Arabia, Turkey, and Iraq had publication bursts in the last 3 years, suggesting that these countries were relatively active in this field during that period with a concentrated number of articles published.

### 3.3 Analysis of the research institution network

The retrieved articles in this study were published by 2,262 institutions. The Chinese Academy of Sciences had the highest number of publications (52) and engaged in active cooperation and communication with other institutions, making it one of the most impactful institutions in the field (Table 2). Other institutions included Fudan University (42), Sichuan University (30), Nanjing University (28), and Sungkyunkwan University (28). The total link strength (TLS) indicates the number of co-occurrences of each institution with other institutions, reflecting their collaboration and communication relationships (Pei et al., 2022). Among all institutions, Sungkyunkwan University in South Korea had the highest TLS score of 63, indicating a strong desire to collaborate with other institutions, followed by the Chinese Academy of Sciences (58), Catholic University Korea (57), and Hanyang University in South Korea (55). Apart from these four institutions, cooperation among other institutions was relatively limited (Supplementary Figure S5), which is unfavorable for the development of this field. The top five institutions, ranked by the average citation per paper, were Seoul National University in South Korea (176.06), the University of Texas MD Anderson Cancer Center (81) in the USA, Nanjing University (63.32), Soochow University (62.63) in China, and Sungkyunkwan University (55.14) in South Korea (Table 2). The Chinese Academy of Sciences, Nanjing University, and Sungkyunkwan University are leaders in this field, as evidenced by their relatively high number of publications and average citations per paper.

**TABLE 2:** The 20 most productive institutions.

Rank	Institutions	Counts	Country	TLS	Total citations	Average citation per paper
1	Chinese Academy of Sciences	52	China	58	2,596	49.92
2	Fudan University	42	China	31	1,421	33.83
3	Sichuan University	30	China	5	736	24.53
4	Nanjing University	28	China	21	1773	63.32
5	Sungkyunkwan University	28	Korea	63	1,544	55.14
6	Tehran University of Medical Sciences	28	Iran	24	1,074	38.36
7	Soochow University	27	China	17	1,691	62.63
8	Zanjan Univ Med Sci	26	Iran	20	769	29.58
9	Sun Yat-sen University	25	China	19	431	17.24
10	Islamic Azad University	24	Iran	22	466	19.42
11	Shanghai Jiao Tong University	24	China	21	621	25.88
12	China Pharmaceutical University	23	China	16	724	31.48
13	Catholic University Korea	22	Korea	57	1,077	48.95
14	Hanyang University	22	Korea	55	632	28.73
15	Univ Texas Md Anderson Canc Ctr	21	United States of America	24	1701	81.00
16	Tarbiat Modares Univ	21	Iran	15	650	30.95
17	Tongji Univ	20	China	9	663	33.15
18	Natl Canc Ctr	18	China	43	496	27.56
19	Univ Chinese Acad Sci	18	China	26	990	55.00
20	Seoul Natl Univ	18	Korea	21	3,169	176.06

### 3.4 Author and co-cited author analysis

The quantity of publications can indicate an author’s level of contribution. The 10 most innovative authors among all authors who published articles related to albumin NPs in the field of oncology are listed in Table 3. Youn YS was the most productive author, with 22 published articles, followed by Choi HG and Lee ES, each with 20 articles.

**TABLE 3:** The top 10 most productive authors.

Rank	Author	Counts	Institutions	H-index	Citations	Average citation per paper
1	Youn YS	22	Sungkyunkwan University	16	1,226	55.73
2	Choi HG	20	Hanyang University	14	625	31.25
3	Lee ES	20	Catholic University Korea	15	957	47.85
4	Danafar H	19	Zanjan University of Medical Sciences	9	619	32.58
5	Dinarvand R	17	Tehran University of Medical Sciences	12	579	34.06
6	Atyabi F	15	Tehran University of Medical Sciences	12	861	57.40
7	Oh KT	15	Chung Ang University	12	547	36.47
8	Shojaosadati SA	14	Tarbiat Modares University	8	400	28.57
9	Hu Y	13	Nanjing University	12	821	63.15
10	Wu J	13	Nanjing University	11	783	60.23

The co-authorship network map (Supplementary Figure S6A) shows that among the top 10 most productive authors, Youn YS collaborated the most with other authors, mainly with Choi HG, Lee ES, and Oh KT, forming one team. Dinarvand R and Atyabi F collaborated closely and formed another team. Hu Y and Wu J collaborated closely and formed a third team. Among the three teams, the Youn YS team published the highest number of articles and had the highest H-index, making it an important driving force for research in this field. The co-authorship network map also revealed a lack of collaboration and communication among the teams.

The co-cited author network map is shown in Supplementary Figure S6B. A higher number of co-citations suggests that the author is more recognized in the field. The three authors with the highest number of co-citations are Kratz F (300 citations), Elzoghby AO (288 citations), and Desai N (269 citations), which indicates these three authors had the greatest academic impact on the application of albumin NPs in oncology (Table 4). Notably, Desai N was involved in the development and application of Abraxane^®^ and Fyarro^®^, which are the only two albumin-bound anti-tumor drugs used in clinic (Ibrahim et al., 2005; Wagner et al., 2021). Therefore, Desai N is a pioneer in the application of albumin-based anti-tumor nanomedicine to clinical practice.

**TABLE 4:** The top 10 most productive co-cited authors.

Rank	Co-cited author	Citations	Centrality
1	Kratz F	300	1.08
2	Elzoghby AO	288	0.42
3	Desai N	269	0.32
4	Gradishar WJ	261	0.13
5	Langer K	205	0.86
6	Weber C	139	0.13
7	Jemal A	136	0
8	Chen Q	132	0.48
9	Von Hoff DD	126	0.11
10	Socinski MA	116	0.02

### 3.5 Journal publication analysis

An analysis of publication journals can assist researchers in quickly and accurately identifying current authoritative journals in the field. Table 5 shows the 10 most productive journals that published 363 articles, accounting for 22.35% of all articles published in this field. The *International Journal of Nanomedicine* published the highest number of articles in this field (64 articles) and had an impact factor (IF) of 8.0 in 2022. It was followed by the *International Journal of Pharmaceutics* (58 articles) with an IF of 5.8 and *Journal of Controlled Release* (40 articles) with an IF of 10.8. Among the 10 most productive journals, *Biomaterials* had the highest IF in 2022 (14.0), the *International Journal of Pharmaceutics* had the highest H-index (30), and the *Journal of Controlled Release* had the highest number of citations (5,762 citations). Furthermore, four of the top 10 journals were ranked Q1 (30%), whereas five were ranked Q2 (50%), indicating that the research content in this field is of relatively high quality.

**TABLE 5:** The top 10 journals.

Rank	Journal	Countries	Count	IF (2022)	JCR (2022)	H-index	Total citations	Percentage
1	International Journal Of Nanomedicine	New Zealand	64	8	Q2	29	4,878	3.94
2	International Journal Of Pharmaceutics	Netherlands	58	5.8	Q2	30	2,805	3.57
3	Journal Of Controlled Release	Netherlands	40	10.8	Q1	27	5,762	2.46
4	Colloids And Surfaces B-Biointerfaces	Netherlands	39	5.8	Q2	20	923	2.40
5	Acs Applied Materials & Interfaces	United States of America	35	9.5	Q2	21	1,321	2.16
6	Journal Of Drug Delivery Science And Technology	France	33	5	Q3	9	270	2.03
7	International Journal Of Biological Macromolecules	Netherlands	26	8.2	Q1	15	808	1.60
8	Biomaterials	Netherlands	24	14	Q1	21	2,250	1.48
9	Journal Of Materials Chemistry B	England	22	7	Q2	16	687	1.35
10	Pharmaceutics	Switzerland	22	5.4	Q3	10	504	1.35

As shown in Table 6, 50% (5/10) of the top 10 co-cited journals were from the USA, and the other 50% (5/10) were from the Netherlands. The *Journal of Controlled Release* had the most co-citations (3,326 citations), with an IF of 10.8 in 2022. The second and third highest co-citations were found in the *Journal of Clinical Oncology* (2,423 citations, IF 45.3) and *Biomaterials* (2,360 citations, IF 14.0), respectively. The *Journal of Clinical Oncology* had the highest IF (45.3) among the top 10 co-cited journals.

**TABLE 6:** The top 10 co-cited journals.

Rank	Co-cited journal	Countries	Citations	IF (2022)	JCR (2022)
1	Journal Of Controlled Release	Netherlands	3,326	10.8	Q1
2	Journal Of Clinical Oncology	United States of America	2,423	45.3	Q1
3	Biomaterials	Netherlands	2,360	14	Q1
4	International Journal Of Pharmaceutics	Netherlands	2009	5.8	Q2
5	International Journal Of Nanomedicine	Netherlands	1,487	8	Q2
6	Acs Nano	United States of America	1,480	17.1	Q1
7	Advanced Drug Delivery Reviews	Netherlands	1,274	16.1	Q1
8	Clinical Cancer Research	United States of America	1,185	11.5	Q1
9	Cancer Research	United States of America	1,128	11.2	Q1
10	Advanced Materials	United States of America	948	29.4	Q1

The trends in the annual publication volume of the top 10 journals on the application of albumin NPs in oncology from 1 January 2000, to 15 April 2024 are shown in Supplementary Figure S7A. The number of publications in the top 10 journals continues to increase. The *International Journal of Nanomedicine* and *International Journal of Pharmaceutics* were the most productive compared with other journals, indicating a greater focus on publishing articles related to this field.

The dual-map overlays of the journals is shown in Supplementary Figure S7B. The figure is divided into two parts: the left side shows the citing journals, and the right side shows the cited journals. The results indicate the position of albumin NPs research in oncology relative to other major research disciplines. Each point on the map represents a journal, and the lines connecting the left and right sides of the map represent citation links. The trajectories of these links provide an understanding of the interdisciplinary relationships within the field and offer a complete picture of the citations. Four main citation paths were notable, indicating that the research on albumin NPs in oncology mainly focused on journals in the fields of Physics, Materials, Chemistry, Molecular, Biology, and Immunology, whereas the cited articles were mainly from journals in the fields of Chemistry, Materials, Physics, Molecular, Biology, and Genetics.

### 3.6 Citation analysis

The top 10 most cited and co-cited articles on the use of albumin NPs in oncology are listed in Tables 7, 8. The article by Schmid P et al. had the highest number of citations (2,675 citations) and ranked first in the average number of citations per year (445.83). As the total number of citations tends to increase with time, earlier publications are more likely to be cited. Therefore, we calculated the average number of citations per year. The article by Schmid P et al. ranked first in both the number of total citations and average citations per year, indicating that the research on immunotherapy in the field of albumin NPs in oncology was an important topic for researchers. This study demonstrated that atezolizumab plus nab-paclitaxel combination therapy prolonged progression-free survival among patients with metastatic triple-negative breast cancer (TNBC) in both the intention-to-treat population and the PD-L1-positive subgroup, and it exhibited good safety (Schmid et al., 2018). The article by De Jong WH et al. ranked second in the total number of citations (2,593) and third in the average number of citations per year (162.06). The article provided a review on the application and potential toxicity of drug-loaded NP technology, emphasizing the need for researchers to consider not only the benefits of nano-drug delivery preparations, such as reducing drug toxicity and side effects and improving therapeutic efficacy, but also how to conduct safety assessments on these preparations (De Jong and Borm, 2008). The study conducted by Paz-Ares L et al. ranked third in the total number of citations (2,353) but ranked second in the average number of citations per year (392.17). The article demonstrated that the addition of pembrolizumab to either the carboplatin-plus-paclitaxel or nab-paclitaxel combination chemotherapy regimen resulted in significantly longer overall survival and progression-free survival (Paz-Ares et al., 2018). This article shows that research on clinical applications is more likely to attract the attention of scholars.

**TABLE 7:** The top 10 articles with the most citations.

Rank	Title	First author	Journal	Year	Total citations	Average citations per year
1	Atezolizumab and Nab-Paclitaxel in Advanced Triple-Negative Breast Cancer	Schmid P (Schmid et al., 2018)	New england journal of medicine	2018	2,675	445.83
2	Drug delivery and nanoparticles: Applications and hazards	De Jong WH (De Jong and Borm, 2008)	International journal of nanomedicine	2008	2,593	162.06
3	Pembrolizumab plus Chemotherapy for Squamous Non-Small-Cell Lung Cancer	Paz-Ares L (Paz-Ares et al., 2018)	New england journal of medicine	2018	2,353	392.17
4	Albumin as a drug carrier: Design of prodrugs, drug conjugates and nanoparticles	Kratz F (Kratz, 2008)	Journal of controlled release	2008	1734	108.38
5	Phase III trial of nanoparticle albumin-bound paclitaxel compared with polyethylated castor oil-based paclitaxel in women with breast cancer	Gradishar WJ (Gradishar et al., 2005)	Journal of clinical oncology	2005	1,590	83.68
6	Pancreatic cancer	Kamisawa T (Kamisawa et al., 2016)	Lancet	2016	1,176	147.00
7	Liposomes and nanoparticles: nanosized vehicles for drug delivery in cancer	Malam Y (Malam et al., 2009)	Trends in pharmacological sciences	2009	970	64.67
8	Nanoparticle-liver interactions: Cellular uptake and hepatobiliary elimination	Zhang YN (Zhang et al., 2016)	Journal of controlled release	2016	785	98.13
9	Milk proteins as vehicles for bioactives	Livney YD (Livney, 2010)	Current opinion in colloid and interface science	2010	683	48.79
10	Impact of albumin on drug delivery-New applications on the horizon	Elsadek B (Elsadek and Kratz, 2012)	Journal of controlled release	2012	658	54.83

**TABLE 8:** The top 10 co-cited references.

Rank	Title	First author	Journal	IF (2022)	JCR (2022)	Year	Citations
1	Albumin-based nanoparticles as potential controlled release drug delivery systems	Elzoghby AO (Elzoghby et al., 2012)	Journal Of Controlled Release	10.8	Q1	2012	92
2	Increased survival in pancreatic cancer with nab-paclitaxel plus gemcitabine	Von Hoff DD (Von Hoff et al., 2013)	New England Journal of Medicine	158.5	Q1	2013	52
3	Strategies for Preparing Albumin-based Nanoparticles for Multifunctional Bioimaging and Drug Delivery	An FF (An and Zhang, 2017)	Theranostics	12.4	Q1	2017	46
4	Weekly nab-paclitaxel in combination with carboplatin *versus* solvent-based paclitaxel plus carboplatin as first-line therapy in patients with advanced non-small-cell lung cancer: final results of a phase III tria	Socinski MA (Socinski et al., 2012)	Journal of Clinical Oncology	45.3	Q1	2012	45
5	Impact of albumin on drug delivery-new applications on the horizon	Elsadek B (Elsadek and Kratz, 2012)	Journal Of Controlled Release	10.8	Q1	2012	43
6	Harnessing albumin as a carrier for cancer therapies	Hoogenboezem EN (Hoogenboezem and Duvall, 2018)	Advanced Drug Delivery Reviews	16.1	Q1	2018	42
7	Nanoparticles for Biomimetic Drug Delivery via Albumin-Binding Protein Pathways for Antiglioma Therapy	Lin TT (Lin et al., 2016)	Acs Nano	17.1	Q1	2016	36
8	Doxorubicin-loaded human serum albumin nanoparticles surface-modified with TNF-related apoptosis-inducing ligand and transferrin for targeting multiple tumor types	Bae S (Bae et al., 2012)	Biomaterials	14	Q1	2012	34
9	The Uniqueness of Albumin as a Carrier in Nanodrug Delivery	Spada A (Spada et al., 2021)	Molecular Pharmaceutics	4.9	Q2	2021	34
10	Albumin as a drug carrier: design of prodrugs, drug conjugates and nanoparticles	Kratz F (Kratz, 2008)	Journal Of Controlled Release	10.8	Q1	2008	33

**FIGURE 2:**
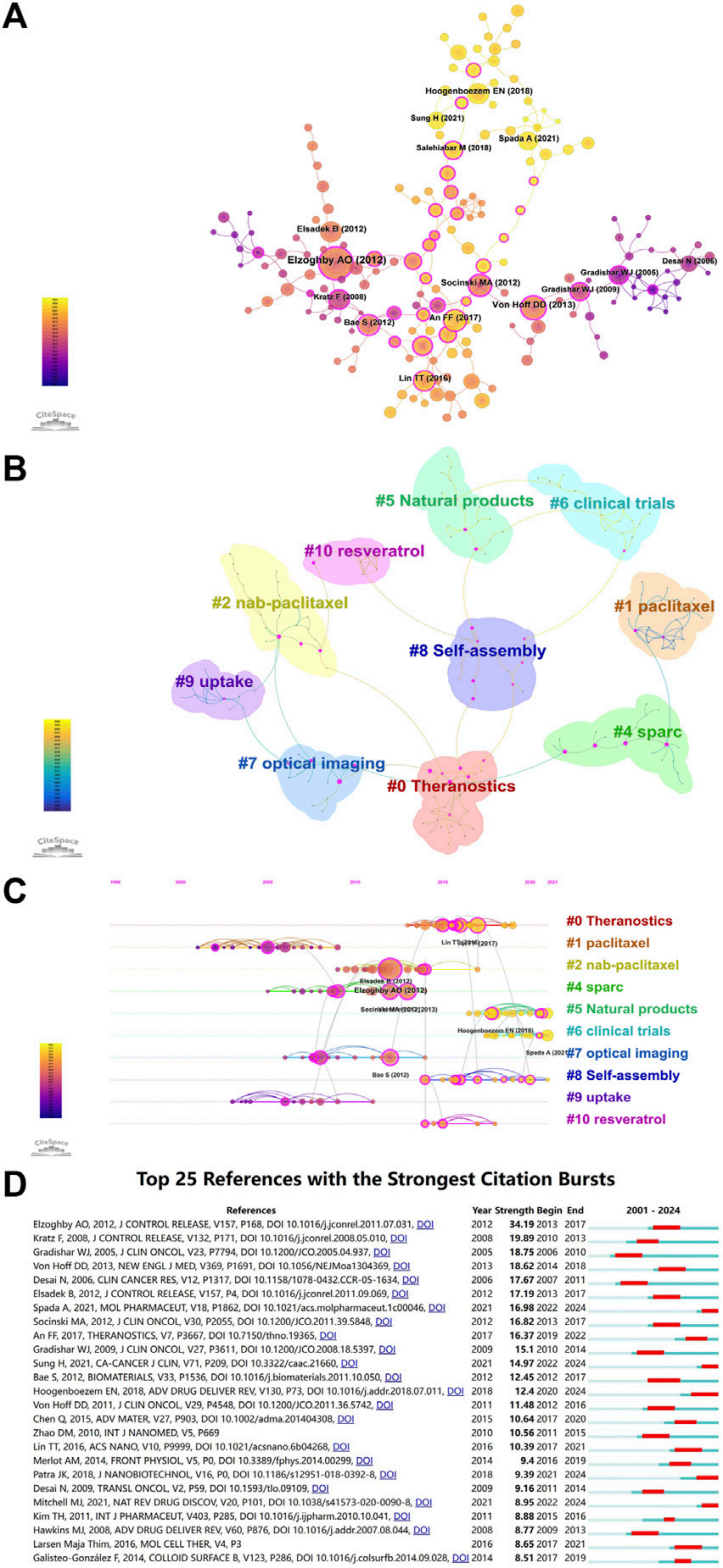
**(A)** Co-citation network of references. The size of the circle is positively correlated with the number of citations. Purple indicates earlier citations and yellow indicates more recent citations. The overlapping colors indicate that the article was cited in all corresponding years. The lines connecting the circles represent co-cited articles. The rose red nodes are the key nodes. **(B)** Cluster analysis of co-cited references. The different colors mean different clusters. **(C)** Timeline distribution of co-cited references. The size of the circle is proportional to the number of co-citations. The line between the articles represents co-citation, i.e., co-cited articles. Purple indicates earlier co-citations, and yellow indicates more recent co-citations. Overlapping colors indicate the article was co-cited in all the corresponding years. Nodes in rose red are nodes with relatively strong centrality (i.e., occupies a central position and serves as a hub). Co-cited articles in the same cluster are positioned along the same horizontal line. The timeline for the occurrence of co-cited articles is placed at the top of the visualization map, with the right representing more recent periods. **(D)** The top 25 References with the Strongest Citation Bursts.

CiteSpace was used to perform co-citation analysis (Figure 2A), involving various data mining tools to analyze the cited articles. The results can provide researchers with multifaceted information, including the ability to identify articles with far-reaching impact in each field. As shown in Figure 2A, the most influential co-cited article was that of Elzoghby AO et al., with 92 co-citations (Table 8). The article provided a detailed discussion of the various types of albumin NPs, their preparation methods, and the main *in vivo* and *in vitro* research findings. Furthermore, it highlighted the potential of albumin NPs in targeted drug delivery to specific tumor sites (Elzoghby et al., 2012).

Next, cluster co-cited analysis was performed based on co-citation analysis (Figure 2B). CiteSpace provides modularity (Q value) and mean silhouette (S value) to assess the clarity and reasonableness of the network structure and clusters. A Q value > 0.3 indicates a significant cluster structure, whereas an S value > 0.5 indicates reasonable clustering. The Q value of Figure 2B is 0.8469 (>0.3), and the S value is 0.961 (>0.5), indicating significant cluster structures and meaningful clustering of the keywords in the sample. Figure 2B also displays that the co-cited articles formed 10 clusters (few nodes in #3 are of no research significance and have been automatically filtered out by CiteSpace), with the top three being: #0 theranostics, #1 paclitaxel, and #2 nab-paclitaxel. The number of articles in each cluster is negatively correlated with the cluster number. For example, cluster #0 contains the highest number of articles. We performed a timeline analysis of the co-cited articles based on their clustering to elucidate the changes in research hotspots over time (Figure 2C). The vertical axis in the figure represents the cluster labels (10 clusters), and the horizontal axis represents the time at which the key reference nodes appeared. The figure illustrates that paclitaxel, uptake, and SPARC (secreted protein acidic and rich in cysteine) were the earliest hotspots (#1, #9, and #4, respectively), followed by theranostics (#0), natural products (#5), clinical trials (#6), and self-assembly (#8), which appeared later, first occurring in 2013, 2017, 2018, and 2014, respectively. However, these fields have received growing interest in recent years and are now considered research hotspots.

The application of albumin NPs in oncology from 1 January 2000, to 15 April 2024 was analyzed using CiteSpace to identify the top 25 citation bursts. A citation burst is defined as a surge in the citations of a given article within a specific period. The article by Elzoghby AO et al. had the strongest citation burst, with the burst period occurring in 2013–2017 (Figure 2D). This article was also the most influential co-cited article (Table 8). The first article to undergo a citation burst (in 2005) was by Gradishar WJ et al., which reported the results of a phase III clinical trial of Abraxane^®^, demonstrating greater efficacy and a better safety profile than standard paclitaxel based on polyethylated castor oil.

Recent highly cited articles (2021–2024) undergoing bursts were published by Spada A et al., Sung H et al., Hoogenboezem EN et al., Patra JK et al., and Mitchell MJ et al. (Figure 2D). The article by Spada A et al. described the advantages of endogenous substances such as albumin as drug delivery carriers, along with the importance of drug carriers to drug properties in biological systems and the possible future application trends of drug delivery systems (Spada et al., 2021). The article by Sung H et al. provided an update on the global cancer burden using the GLOBOCAN 2020 estimates of cancer incidence and mortality projected by the International Agency for Research on Cancer (Sung et al., 2021). Hoogenboezem EN et al. systematically reviewed the advantages of albumin as an anti-tumor drug carrier, drug–albumin binding methods, loadable drugs, and the results of *in vivo* and *in vitro* studies. In addition, the paper also highlighted that characterizing how different classes of cargo and types of albumin engagement affect systemic and intracellular trafficking will be critical for optimizing future therapeutic candidates (Hoogenboezem and Duvall, 2018). The review by Patra JK et al. summarized the recent progress and development prospects in the field of nanomedicines and nano-based drug delivery systems and highlighted the opportunities and challenges of developing nanomedicines from synthetic and natural sources and their clinical applications (Patra et al., 2018). Mitchell MJ et al. demonstrated advances in NP design that overcome heterogeneous barriers to delivery, arguing that intelligent NP design can improve overall patient outcomes by improving efficacy in general delivery applications while enabling tailored designs for precision applications (Mitchell et al., 2021).

### 3.7 Hotspot word frequency analysis

Keywords are a brief expression of an article’s primary theme and content. Related keywords that appear in the same article exhibit keyword co-occurrence, and the number of co-occurrences can indicate their proximity. A keyword co-occurrence network represents the research focus and connections within a field. By analyzing the keywords, we can clarify the hotspots and frontiers of the research field and predict future developments.

Based on the top 20 most frequently used keywords (Table 9), the most investigated drugs in this field were nab-paclitaxel, paclitaxel, doxorubicin, curcumin, and gemcitabine. The most investigated tumors were breast cancer and pancreatic cancer, and the most investigated carrier protein was HSA. However, HSA has a limited yield and is costly to produce owing to its derivation from human blood. Therefore, BSA has also been widely investigated because of its similar amino acid composition, molecular weight, and spatial structure to those of HSA. Furthermore, it is more widely available and cost-effective than HSA. However, BSA is an exogenous macromolecule that can induce allergic reactions in humans, limiting its use mainly to animal studies (Voltolini et al., 2013). In contrast, HSA is non-immunogenic as it is derived from humans. Therefore, it is extensively used in clinical trials as a material in the final product. The main treatment method adopted in the studies was chemotherapy. Another focus of research in this field was photothermal therapy based on albumin NPs.

**TABLE 9:** The top 20 keywords.

Rank	Keywords	Frequency	TLS	Rank	Keywords	Frequency	TLS
1	nanoparticles	293	694	11	cancer	57	145
2	nab-paclitaxel	217	392	12	pancreatic cancer	55	112
3	drug delivery	155	340	13	doxorubicin	49	107
4	human serum albumin	143	360	14	nanomedicine	48	124
5	albumin	124	298	15	curcumin	47	117
6	albumin nanoparticles	123	206	16	folic acid	42	111
7	paclitaxel	122	269	17	gemcitabine	42	98
8	bovine serum albumin	98	199	18	targeted drug delivery	40	93
9	breast cancer	93	216	19	photothermal therapy	39	84
10	chemotherapy	78	184	20	cancer therapy	38	85

**FIGURE 3:**
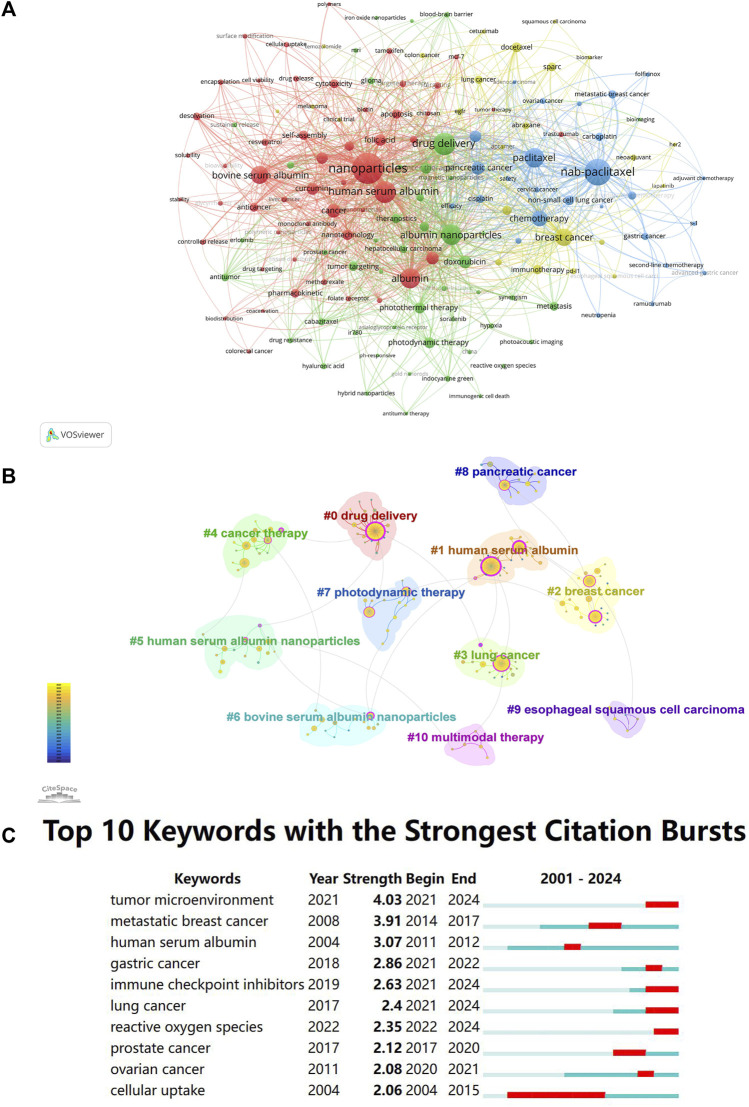
**(A)** Clustering network of keywords by VOSviewer. Each circle and label form one node. The size of the circle is positively correlated with the frequency of keywords. The thickness of the line connecting the circles indicates the strength of the positive correlation between keywords. Nodes of different colors form different clusters, and different colors represent different research directions. **(B)** Clustering network of keywords by CiteSpace. **(C)** The top 25 keywords with the strongest citation bursts.

Using VOSviewer, co-occurrence analysis was conducted on the article keywords, with a minimum of three occurrences for each keyword. The analysis resulted in four clusters (Figure 3A), which can be summarized as the following four themes: “Comprehensive Evaluation,” “Multifunctional NPs and Combination Therapy,” “Nab-paclitaxel,” and “Clinical Application.” The red cluster represents comprehensive evaluation, which involves constructing, characterizing, and evaluating drug-loaded albumin NPs *in vivo* and *in vitro*. The keywords in this cluster included “HSA,” “BSA,” “self-assembly,” “curcumin,” and “folic acid.” The green cluster represents the study on combination therapy based on albumin nanotechnology and the construction of multifunctional albumin NPs. The keywords in this cluster included “photothermal therapy,” “theranostics,” “photodynamic therapy,” “combination therapy,” and “doxorubicin.” The blue cluster represents the topic “nab-paclitaxel,” which involves the study of the application of nab-paclitaxel monotherapy and combination therapy in different types of tumors. The keywords included in this cluster included “nab-paclitaxel,” “non-small cell lung cancer,” “carboplatin,” “pancreatic cancer,” and “gemcitabine.” The yellow cluster represents studies on clinical application, focusing on the clinical research of various anti-tumor treatment options based on albumin NPs. The keywords in this cluster included “breast cancer,” “docetaxel,” “immunotherapy,” “lung cancer,” and “clinical trial.”; Keywords were clustered by CiteSpace, and a total of 11 clusters were obtained (Figure 3B). The Q value of Figure 3B was 0.8256 (>0.3), and the S value was 0.9643 (>0.5), indicating significant cluster structures and meaningful clustering of the keywords in the sample. As shown in Figure 3B, these keywords formed 11 clusters: #0 drug delivery, #1 human serum albumin, #5 human serum albumin nanoparticles, and #6 bovine serum albumin nanoparticles can be classified under comprehensive evaluation; #2 breast cancer, #3 lung cancer, #4 cancer therapy, #8 pancreatic cancer, and #9 esophageal squamous cell carcinoma can be classified under clinical application; and #7 photodynamic therapy and #10 multimodal therapy can be classified under multifunctional NPs and combination therapy. These classifications were generally consistent with those of VOSviewer.

The term “burst map” refers to the sudden increase in the frequency of a particular keyword within a specific time frame. The top 10 keywords with the strongest bursts between 1 January 2000, and 15 April 2024 in the field of albumin NPs in oncology are shown in Figure 3C. The red area in the figure indicates the burst period for each keyword. The most popular keywords from 2022 to 2024 were “tumor microenvironment,” “gastric cancer,” “immune checkpoint inhibitors,” “lung cancer,” and “reactive oxygen species.” The burst of tumor microenvironment (TME) and immune checkpoint inhibitors suggests that albumin NP-based tumor immunotherapy is a current hotspot. Tumor immunotherapy is an emerging treatment method that activates the immune system to fight tumors and exhibits the advantages of long-lasting efficacy, few side effects, and wide application. Conversely, the traditional drug delivery method has poor targeting and a short residence time in TME, which limit the therapeutic effect. The albumin NPs effectively penetrate the TME and specifically deliver immunotherapy drugs to the TME for a certain period, which improves the therapeutic effect (Yang et al., 2021). Reactive oxygen species (ROS) refers to a class of oxygen-containing substances that are directly or indirectly converted from oxygen molecules and have more active chemical reactivity than oxygen molecules. They have multiple functions such as killing tumors, inhibiting chemoresistance, and inducing systemic immunity (Bhushan and Gopinath, 2015). The burst of the keyword “reactive oxygen species” indicates that these ROS-based anti-tumor therapies (such as chemodynamic, photodynamic, and sonodynamic therapies) are hot topics in the field of albumin NPs in oncology. Similarly, bursts of the keywords “gastric cancer” and “lung cancer” indicate that these tumors have attracted much attention from researchers in recent years.

## 4 Discussion

### 4.1 Overview of research

The research indicates a growing trend in the use of albumin NPs as anti-tumor drug carriers over the past 20 years, with a notable increase in publications after 2015 suggesting a continued interest in this topic. Considering the numerous advantages of albumin NPs as drug carriers, including biodegradability, biocompatibility, non-immunogenicity, and long half-life, as well as the successful launch of Abraxane^®^ and Fyarro^®^, albumin NPs hold great promise in the targeted delivery of anti-tumor drugs. After analyzing research regions, we found that China and the USA published a significantly higher number of articles than other countries/regions and these were the two most productive countries in this field. However, the average citation frequency of Chinese articles was low, ranking eighth among the top 10 countries. These findings suggest that, despite the large number of articles published in China, the quality of their articles is relatively low. Therefore, researchers should be guided and encouraged to conduct innovative research. Quantity alone is not meaningful without high-quality research results. Furthermore, when evaluating scientific research performance, quantitative evaluation indicators cannot replace research evaluation. Instead, a quality-oriented evaluation system should be actively explored to promote the high-quality development of scientific research. The USA has a dominant presence in this field, as evidenced by high citations, H-index, average citations per paper, and TLS. These objective metrics indicate the impact of the articles from the United States.

Eleven of the 20 most productive institutions in this field were from China, which ranked first in productivity. This indicates that Chinese researchers prioritize research in this field and have invested substantial research effort, resulting in China having the highest number of publications. Researcher network analysis revealed that four authors from four South Korean institutions (Youn YS, Choi HG, Lee ES, and Oh KT) formed one team and published the highest number of articles with the highest H-index, making them an important driving force for research in this field. Kratz F, Elzoghby AO, and Desai N were identified as co-cited authors with considerable academic influence in this field. Desai N was involved in the development and application of Abraxane^®^ and Fyarro^®^ and is a pioneer in the application of albumin-bound anti-tumor drugs to clinical practice.

The *International Journal of Nanomedicine* and the *International Journal of Pharmaceutics* had the highest number of publications in this field, indicating that these two journals focus on the publication of articles in this field. Therefore, researchers in this field can be recommended to contribute to these journals. The *Journal of Controlled Release* had the highest number of co-citations, whereas the *Journal of Clinical Oncology* had the highest IF among the co-cited journals. For researchers to gain a better understanding of research advances in this field, they should read articles published by the authors or journals mentioned above. Furthermore, these high-impact authors may serve as potential collaborators. However, collaboration among different countries, institutions, and teams is limited, particularly among those from different countries, which hinders this aspect of research. Therefore, we recommend that researchers improve collaboration, mutual learning, and communication.

### 4.2 Research hotspots and frontiers

Co-citation analysis is the use of data mining tools to analyze cited articles, authors, and journals. The results provide researchers with multifaceted information, including the identification of authors with far-reaching impact within the field, research teams who are active within the academic community, and trends in research topics, thereby facilitating better recognition of important research directions and future research hotspots.

Based on the citation analysis, we found that in addition to review articles, three articles by Schmid, P et al., Paz-Ares et al., and Von Hoff DD et al. were heavily cited or co-cited. These three articles were all clinical studies on the combination therapy of nab-paclitaxel, which indicates that this topic is of great concern to researchers. The combination therapy regimens discussed in these articles were all related to nab-paclitaxel plus immune checkpoint inhibition, indicating that research on immune checkpoint inhibitors is also a hot research direction in this field. Cluster analysis was used to identify content in the knowledge map network and facilitate the clear extraction of popular topics within the research field. The cluster timeline of co-cited articles revealed that theranostics, natural products, clinical trials, and self-assembly have received growing attention in recent years. Through the analysis of keyword co-occurrence, we found that research on albumin NPs mainly focuses on comprehensive evaluation, multifunctional NPs, combination therapy, nab-paclitaxel, and clinical application. The analysis of high-frequency keywords revealed that the most investigated drugs were paclitaxel, doxorubicin, curcumin, and gemcitabine. The most investigated tumors were breast cancer and pancreatic cancer. The most widely investigated carrier proteins were HSA and BSA. Citation and keyword burst maps reflect research frontiers, hotspots, and growing trends within a field. Notably, according to citation and keyword burst maps, articles on TME, gastric cancer, immune checkpoint inhibitors, lung cancer, and ROS have been extensively cited in recent years, and these topics may become research hotspots.

Based on the results of our analysis of article co-citation, clusters, bursts, keyword co-citation, high-frequency keywords, cluster labels, and our cognition in this field, we suggest that the six categories mentioned in the following text may become future research hotspots and frontiers.

#### 4.2.1 Rapid and convenient synthesis methods predominated by self-assembly

Various methods have been developed for preparing albumin NPs, including thermal gelation (Qi et al., 2010), nano-spray drying (Arpagaus et al., 2018), desolvation (Jahanban-Esfahlan et al., 2016), emulsification (Zu et al., 2013), Nab^TM^ technology (Wan et al., 2015), and self-assembly (Safavi et al., 2017) (Table 10). Thermal gelation is a simple and high-yield method that produces NPs with good uniformity. However, it requires a high preparation temperature that is unsuitable for encapsulating unstable drugs. Nano-spray drying enables the production of NPs with high uniformity and encapsulation efficiency, making them capable of encapsulating hydrophilic drugs. However, this method has strict preparation requirements, long preparation times and produces NPs with a relatively large particle size. Desolvation is a commonly used method for preparing albumin nanocarriers owing to its simplicity and rapidity, despite the need for the addition of organic solvents or cross-linking agents, which can be relatively toxic (Loureiro et al., 2016). Another simple and cost-effective method is emulsification. However, the emulsification process can disrupt the stability of albumin, making it difficult to control the microscopic morphology of the NPs and resulting in poor reproducibility and high batch-to-batch variation. Furthermore, like desolvation, the preparation process involves the use of organic solvents or cross-linking agents, which can be relatively toxic.

**TABLE 10:** Overview of albumin nanoparticle synthesis strategies.

Method	Description	Schematic representation	Application
Thermal gelation	Heat induces the unfolding of albumin molecules, and the interactions among albumin molecules result in their aggregation to form stable NPs	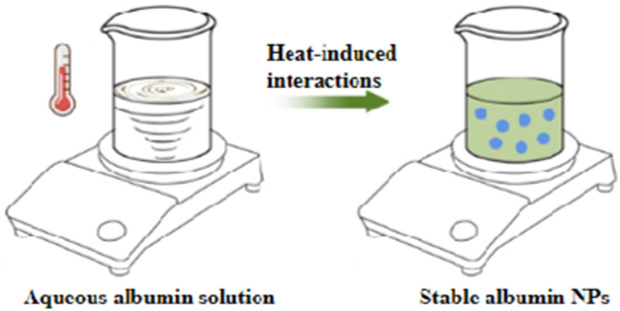	Doxorubicin-loaded BSA–dextran–chitosan NPs (Arpagaus et al., 2018)
Nano-spray drying	The drug solution is first sprayed to form nanodroplets, which are then dried by the flow of heated gas (carbon dioxide and nitrogen) into the chamber. Finally, the NPs are collected through electrode induction at the bottom	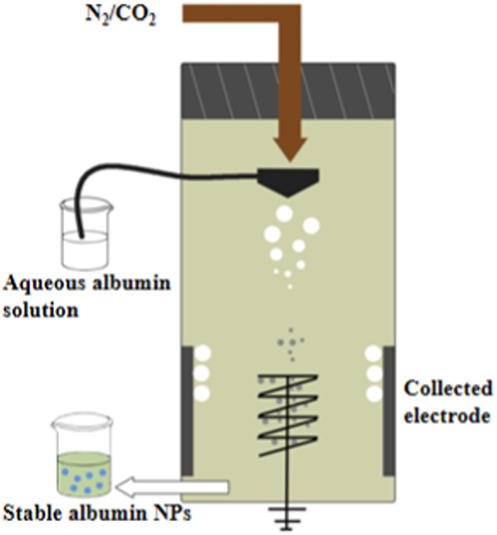	BSA NPs (Lee et al., 2011)
Desolvation	An organic solvent is added under constant stirring to expose the hydrophobic region of albumin. Once the solubility is reduced, the albumin aggregates are precipitated. Then, relatively stable albumin NPs are formed through thermal denaturation or chemical cross-linking	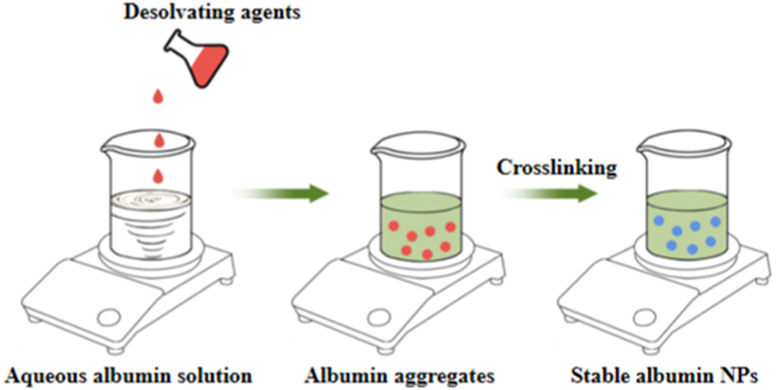	HSA and BSA NPs (Jahanban-Esfahlan et al., 2016)
Emulsification	The aqueous solution containing albumin is mixed with the oil phase containing the drug and emulsifier, followed by emulsification through methods such as stirring, ultrasonic mixing, or high-pressure homogenization to obtain a water-in-oil emulsion. The emulsion is then cured through thermal denaturation or chemical cross-linking to obtain albumin NPs.	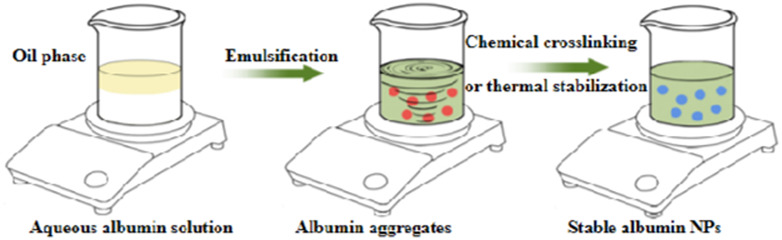	10-hydroxycamptothecin-loaded glycyrrhizic acid-conjugated bovine serum albumin NPs (Zu et al., 2013)
Nab^TM^	The hydrophobic drug is dissolved in a non-polar solvent and added to the aqueous albumin solution. A nanoemulsion is then formed under low-speed shear force, followed by high-pressure homogenization. Finally, the non-polar solvent is removed to obtain stable albumin NPs	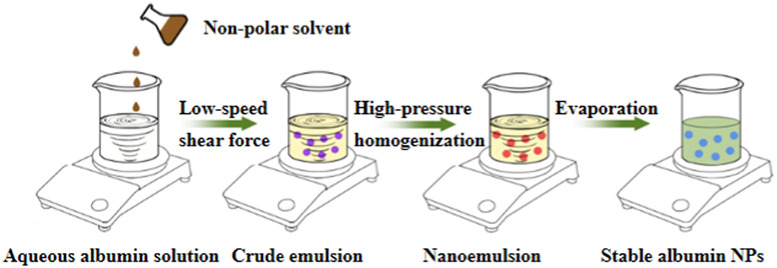	Lapatinib-loaded human serum albumin NPs (Wan et al., 2015)
Self-assembly	The disulfide bonds of albumin are reduced to sulfhydryl groups by adding reductants. Then, denaturants or hydrophobic drugs are added to decrease the number of primary amine groups on the surface of albumin and increase the hydrophobicity of the albumin molecules. This induces the self-assembly of the molecules through hydrophobic associations to form stable NPs	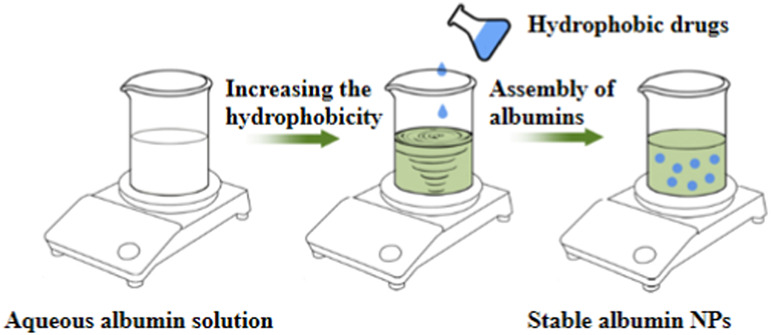	Curcumin-loaded albumin NPs (Safavi et al., 2017)

Nab^TM^ technology uses a self-cross-linking method, eliminating the need for cross-linking agents. The process involves disrupting the disulfide bonds in albumin molecules through heating, reductants, and high pressure to induce homogeneous unfolding. This is followed by the resynthesis of disulfide bonds within and between albumin molecules, resulting in the cross-linking of albumin to form NPs. Notably, only two albumin-bound anti-tumor drugs are currently available worldwide—Abraxane^®^ and Fyarro®—both of which are prepared using Nab^TM^ technology. However, the preparation process still involves the addition of organic solvents, which may lead to issues with solvent residues (Ding et al., 2014). In addition, Nab^TM^ technology requires relatively high equipment standards. Furthermore, albumin aggregation during the homogenization process may cause difficulties in dispersing the product after freeze-drying. Each method listed above has its own advantages and disadvantages, and the appropriate method should be selected based on the properties of the drugs when preparing NPs. Moreover, new preparation technologies are actively being explored in addition to the aforementioned methods. Self-assembly preparation method has gained increasing attention in recent years (Safavi et al., 2017). Similar to Nab^TM^ technology, self-assembly uses the self-cross-linking approach without requiring the highly specialized equipments and organic solvent residues associated with Nab^TM^ technology. Furthermore, self-assembled NPs have small particle sizes and good flexibility, enabling effective penetration of physiological barriers and efficient drug delivery to target tissues or organs. Self-assembly has become a research hotspot in recent years because of its advantages and has been extensively studied and applied (Lin et al., 2016; Liu et al., 2023; Lai et al., 2023). However, this method also has limitations, including the requirement of the use of reductants such as β-mercaptoethanol, dithiothreitol, and cysteine, which pose certain safety risks during preparation. Therefore, further research is necessary to optimize this method.

#### 4.2.2 Surface modification

**FIGURE 4:**
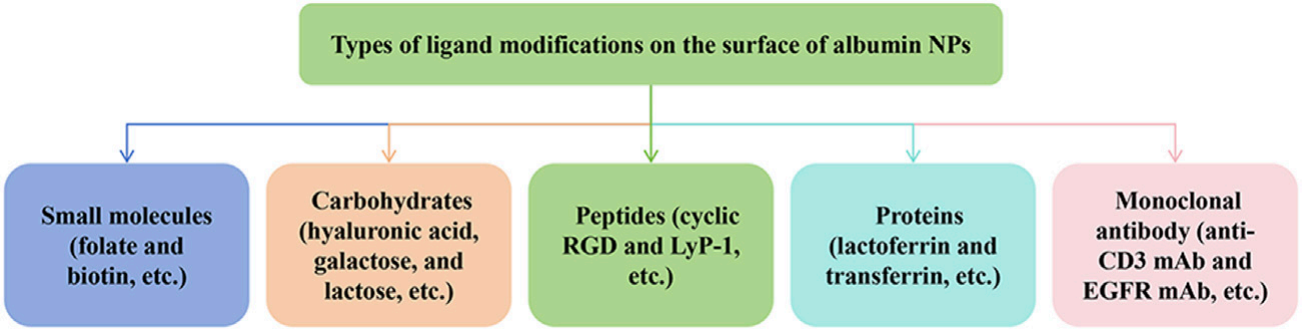
Types of ligand modifications on the surface of albumin NPs.

Owing to the EPR effect and macropinocytosis, albumin can evade the recognition and phagocytosis of the mononuclear phagocyte system and passively target the liver, kidney, bone marrow, and other organs. However, the EPR effect demonstrates great heterogeneity in solid tumors, varying among different tumor types, sizes, sites, patients, and stages of development (Subhan et al., 2023). Therefore, the EPR effect does not guarantee the efficacy and reliability of targeted therapy. To enhance the delivery of chemotherapy drugs to specific tumor sites, researchers usually modify the surface of albumin NPs to achieve active targeting of albumin NPs *in vivo*. The surface-modified albumin NPs enter the blood circulation and the tumor cells by recognizing the highly expressed receptors on the membranes of tumor cells. Compared with the unmodified albumin NPs, surface-modified albumin NPs exhibit a more specific targeting effect and further increase the accumulation of NPs at the tumor site. In tumor tissues, the tight junctions of cells disappear and receptors on the tumor cells are exposed, which mediates the entry of surface-modified NPs into cells. Conversely, the vascular epidermal junctions on the surface of some normal cells are tightly connected, and receptors are not directly exposed to the blood circulation, preventing the entry of the surface-modified NPs in these cells. Common methods for surface modification of albumin NPs include covalent coupling modification, conjugated ligand modification, and antibody modification. Among them, ligand-modified albumin NPs have been more frequently studied. The types of modified ligands currently studied include small molecules (such as folic acid and biotin), carbohydrates (such as hyaluronic acid, galactose, and lactose), peptides (such as cyclic RGD and LyP-1), proteins (such as lactoferrin and transferrin) and monoclonal antibody (Monoclonal antibody (anti-CD3 mAb and EGFR mAb) (Tao et al., 2021) (Figure 4).

Folic acid (FA) is one of the most widely used ligands owing to its high efficiency and low cost. Its receptor is overexpressed on the surface of many human tumors, such as ovarian, lung, breast, and endometrial cancers. The upregulation of folate receptors in tumor tissue leads to lower local toxicity and greater anti-tumor efficacy of folate-related therapeutic drugs compared with those of non-targeted drugs. Therefore, the use of FA to modify albumin nanomedicine increases specific tumor targeting (Bardania et al., 2023). Firouzabadi K et al. used desolvation to synthesize polyethylene glycol-modified albumin NPs loaded with naringenin and bound FA to the NP surface and reported that these NPs exhibited dramatic anti-breast cancer effects (Firouzabadi et al., 2023).

Hyaluronic acid (HA) is another widely used ligand. It has the broadest application prospects and is also a current research hotspot. Albumin and HA have rich functional groups and can be easily combined together. The negatively charged HA in the physiological environment reverses the positive charge on the surface of albumin, avoiding the non-specific adsorption caused by the positive charge and prolonging the half-life of NPs *in vivo*. HA can specifically bind to various receptors that are highly expressed in tumor cells, such as cell surface receptor CD44, in a variety of cancers, including breast, lung, and gastric cancers. In addition, HA, like albumin, is biocompatible, biodegradable, and non-immunogenic, and their combination can better adapt to the *in vivo* environment (Lei et al., 2021; Subhan et al., 2023). Based on these advantages, the combination of HA and albumin has irreplaceable advantages as a nano-carrier for chemotherapy drugs, and some studies have demonstrated their potential and development prospects. Ye M et al. encapsulated the fluorescent tracer boron drug BS-CyP in albumin NPs and modified the NPs with HA to develop a novel tumor-targeting boron NP. Notably, the NPs effectively delayed the release of drugs in tumors, enhanced the accumulation of drugs in tumors, and exhibited good safety with no notable toxicity to cells and mice.

Studies on modified ligands such as glycyrrhizin, lactose, Arg–Gly–Asp peptide, aptamer, and transferrin are extensive and show an increasing trend year over year (Saleh et al., 2019; Stecanella et al., 2021; Zheng et al., 2023). Owing to the role of surface modification in improving the targeting of albumin nanodrugs, prolonging the circulation time *in vivo* and increasing the selective accumulation of drugs at tumor sites may result in broad application prospects in the field of albumin NPs in oncology.

#### 4.2.3 Construction of multifunctional NPs for theranostics

Although numerous diagnosis and treatment methods have been developed, cancer still poses a global threat to human health. The mortality rate of cancer continues to increase each year. Therefore, more effective cancer treatment strategies are needed to achieve more sensitive diagnosis methods and efficacious treatment (Siddique and Chow, 2022). Theranostics based on multifunctional nanomedicine is a powerful strategy that combines diagnostics with therapeutic regimens to overcome the shortcomings of current conventional clinical treatments for cancer (Hoogenboezem and Duvall, 2018). However, a single diagnostic and treatment method is usually insufficient because of the insidiousness and drug resistance of tumors. Therefore, the long-term goal has been to develop multifunctional NPs that combine multiple diagnostic and therapeutic modalities. Albumin NPs have been widely employed in numerous fields, including drug delivery, bioimaging, diagnostics, and therapeutics, based on their unique physical, chemical, and biological properties (An and Zhang, 2017). Albumin NPs have a large specific surface area and numerous groups for chemical modification, making them an excellent platform for designing and constructing multifunctional NP systems for theranostics. Multifunctional albumin NPs can combine magnetic resonance imaging (MRI) with near-infrared fluorescence imaging (NIRFI), enabling the high spatial resolution of MRI to complement the high sensitivity of NIRFI (Liu et al., 2023). In addition, photothermal therapy (PTT) can be combined with photodynamic therapy (PDT) during imaging to overcome the heat shock effect of PTT and the hypoxic TME of PDT, thereby achieving the combination of multiple diagnostic and therapeutic modalities to improve treatment outcomes (Sahu et al., 2016; Liu et al., 2023).

**FIGURE 5:**
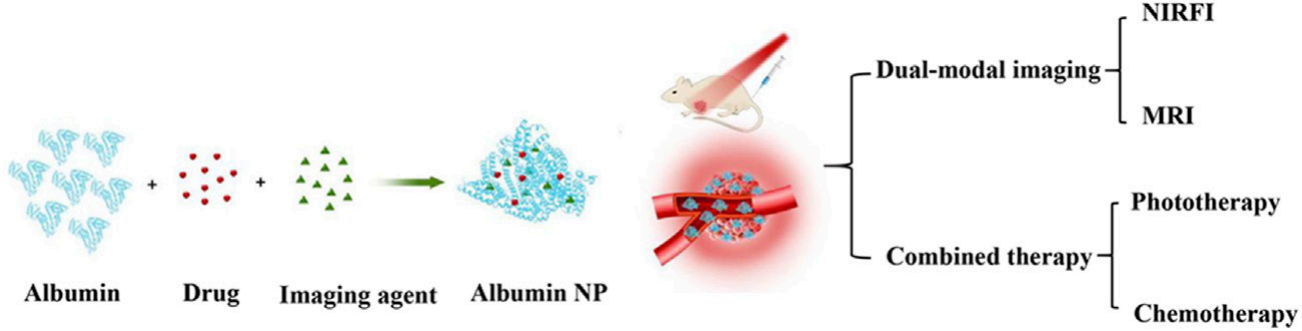
Multifunctional NPs that combine diagnostics and therapeutics.

Heptamethine cyanines with near infrared (NIR) light responsiveness are the most widely studied reagents for bioimaging and diagnosis. Their encapsulation in nanomaterials enables high accumulation at tumor sites, allowing tumor visualization via NIR fluorescence and photoacoustic imaging. After interacting with NIR light, these heptamethine cyanine-incorporating nanomaterials produce photothermal/photodynamic effects with high spatio–temporal resolution and minimal side effects, thereby improving therapeutic outcomes (Leitão et al., 2020). Indocyanine green (ICG) is a star member of the heptamethine cyanine family, as it is one of the few organic dyes approved by the FDA for medical imaging and diagnosis. Besides its bioimaging and diagnostic functions, ICG serves as a multifunctional drug to generate heat or ROS in response to NIR in PTT or PDT (Pham et al., 2020). Loading ICG into albumin NPs can overcome the limitations of free ICG, such as poor stability, a short half-life, and a lack of tissue targeting. Furthermore, it allows for simultaneous application with other co-loaded reagents to achieve imaging-guided combination therapy with multiple therapeutic modalities, including PTT, PDT, thermotherapy, and chemo-PTT, thereby greatly enhancing treatment outcomes (Liu et al., 2018; Kim et al., 2021; Zhong et al., 2021) (Figure 5). However, ICG has low photostability and low fluorescence quantum yield, stimulating the development of other members of the heptamethine cyanine family. IR780 is another heptamethine cyanine reagent that has shown great potential for biomedical applications and drug delivery, and it has been widely used in PTT and PDT because of its high selective uptake by tumor cells and phototoxicity to tumor cells under NIR light irradiation. However, its strong hydrophobicity, poor photostability, poor tolerance, and high toxicity *in vivo* limit its clinical application. The use of albumin NPs as IR780 nanocarriers is an effective strategy to overcome this. Lian et al. co-loaded IR780 and docetaxel (DTX) in albumin NPs for targeted imaging and for PTT/PDT with chemotherapy for castration-resistant prostate cancer treatment. Notably, HSA–IR780–DTX NPs demonstrated significant targeting and therapeutic potential for castration-resistant prostate cancer treatment (Lian et al., 2017).

The construction of multifunctional NPs for theranostics in which albumin NPs act as carriers of magnetic resonance contrast agents is a current research hotspot. Gadolinium-based contrast agents (GBCAs) are mostly used in clinically enhanced MRI. However, GBCAs lack tumor specificity and can cause chronic gadolinium poisoning, limiting their clinical application. Encapsulation of GBCAs in albumin NPs may overcome these challenges (Du et al., 2016). Xuejiao Song et al. used polypyrrole (PPy), Gd (3+)-labeled photosensitizer chlorine e6 (Ce6), and BSA to construct PPy@BSA–Ce6 NPs that exhibited strong tumor aggregation and improved the synergistic therapeutic effects of PDT and PTT (Song et al., 2015). In addition, some metal particles (such as Fe^3+^) can be used as MRI contrast agents and may induce tumor cell apoptosis. Qi Xie et al. used BSA, Fe^3+^-based metal–phenolic networks, and bleomycin (BLM) to construct an all-in-one therapeutic nanoplatform (BFE@BSA NPs) for TNBC with synergistic photothermal and chemodynamic therapy guided by MRI. After entering the body, under laser irradiation, BFE@BSA NPs converted light into heat to ablate tumors. The elevated local temperature accelerated the Fenton reaction initiated by Fe^2+^ (from Fe^3+^ reduction by glutathione) and BLM, further killing tumor cells. Moreover, owing to the presence of Fe^3+^, BFE@BSA NPs can be used as T_1_-weighted MRI contrast agents to provide diagnosis and treatment monitoring for individualized precise therapy (Xie et al., 2022).

Furthermore, in addition to NIR and MRI imaging, computer X-ray tomography and photoacoustic image-guided PDT and PTT therapy for cancer using albumin NPs have also been widely studied in recent years, which may catch the attention of future researchers (Wu et al., 2020).

#### 4.2.4 Research on natural active ingredients mainly based on phenolic compounds

**FIGURE 6:**
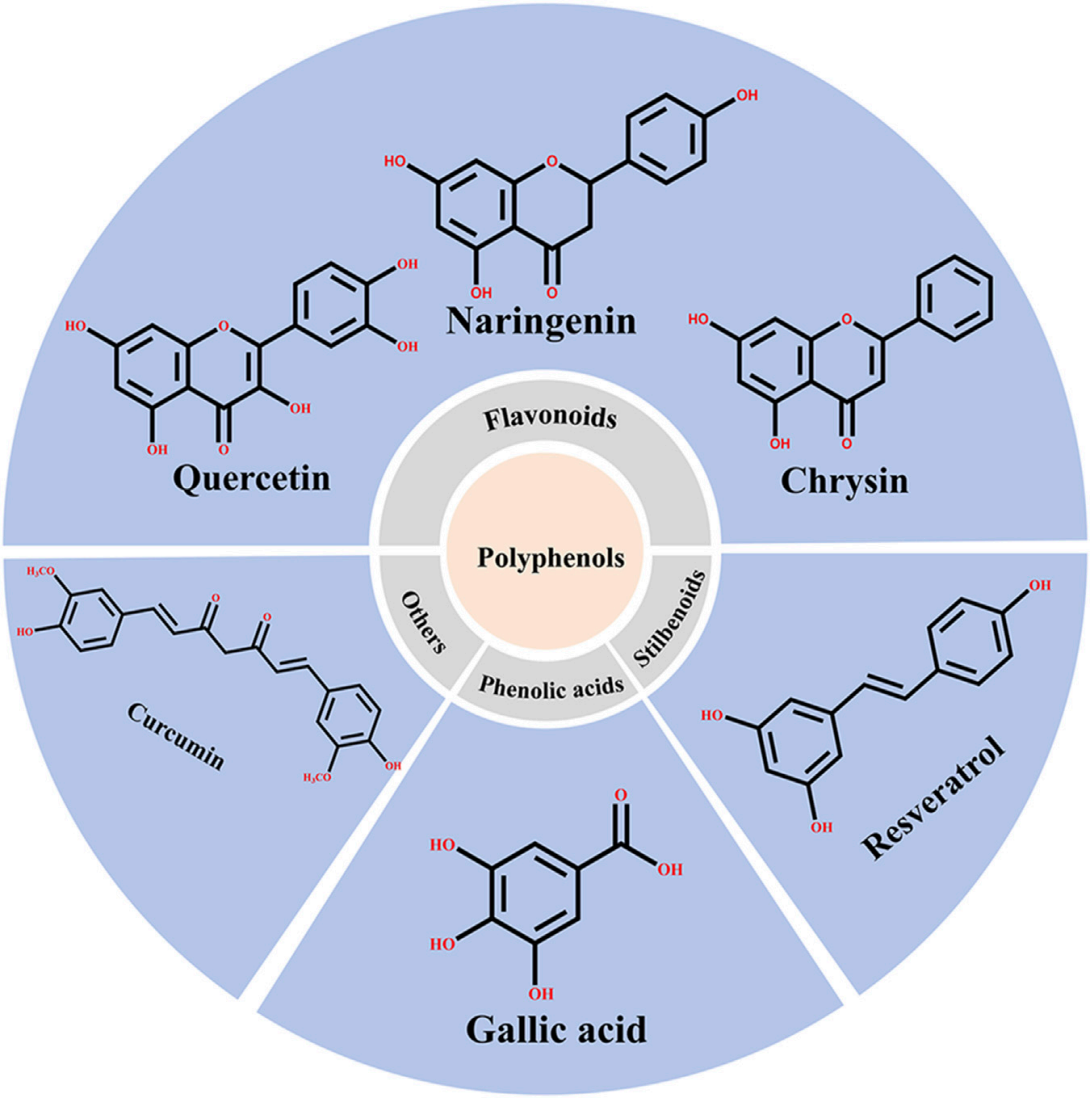
Classification of polyphenolic compounds and their representative compounds in the application of albumin NPs to cancer research.

Natural products with anti-inflammatory, anticancer, and antioxidant properties have been another focus of research. Encapsulation of these compounds using various delivery methods enhances their stability and bioavailability *in vivo*, reduces adverse reactions, and improves targeting, which enhances their anti-tumor effects. One example of an extensively studied and encapsulated natural active ingredient in the application of albumin NPs to oncology is paclitaxel (Chavda et al., 2022). Phenolic compounds, which are widely found in natural plants, are the most studied and encapsulated natural active ingredients in the albumin NPs for oncological application owing to their anti-tumor, anti-inflammatory, and anti-microbial capabilities (Wang et al., 2022). Phenolic compounds can be divided into three main categories: flavonoids, stilbenoids, and phenolic acids. Flavonoids are the largest category of phenolic compounds and are the primary focus of research (Guo et al., 2021), including quercetin (Desale et al., 2018), naringenin (Firouzabadi et al., 2023), diosgenin (Ayatollahi et al., 2022), and chrysin (Ferrado et al., 2019). Furthermore, stilbenoids (mainly resveratrol (Geng et al., 2017; Long et al., 2020; Zhao et al., 2020)) and curcuminoids (mainly curcumin (Kim et al., 2017; Salehiabar et al., 2018; Matloubi and Hassan, 2020)) are the most studied non-flavonoid phenolic compounds in this context, whereas phenolic acids have been studied less frequently. Figure 6 presents the primary classifications of phenolic compounds and their representative compounds in the albumin NPs for oncological application.

The prominent studies on phenolic compounds in the application of albumin NPs to oncology are listed in Table 11. Curcumin is the most investigated phenolic compound, with BSA being the most widely used carrier and self-assembly being the most common preparation method. Curcumin, a natural fatty acid synthase inhibitor, inhibits the proliferation of tumor cells, induces apoptosis of tumor cells, and is widely used in the treatment of breast cancer, lung cancer, pancreatic cancer, and other tumors (Ma et al., 2022). However, the hydrophobicity and instability of curcumin have led to its limited clinical application. Therefore, the development of nano-drug delivery systems for curcumin is particularly important to improve its therapeutic efficiency. In addition, the pharmacological activity of curcumin reduces the side effects of paclitaxel. Accordingly, the co-loading of paclitaxel and curcumin in albumin NPs may exert their combined anti-tumor effect and reduce the side effects of paclitaxel (Ashrafizadeh et al., 2020; Gao et al., 2024). Consequently, curcumin is the most studied phenolic compound in the field of anti-tumor albumin NPs in oncology. In addition, Table 11 shows that although phenolic compounds have been widely used in anti-tumor research, studies on this type of drugs in this field are limited.

**TABLE 11:** Application of phenolic compounds encapsulated by albumin NPs in cancer research.

No.	Bioactive compound	Albumin	Method	Title	Author	Published time
1	Genistein	BSA	Self-assembly	PEGylation of genistein-loaded bovine serum albumin nanoparticles and its effect on *in vitro* cell viability and genotoxicity properties	Ferrado et al. (2023)	2023
2	Genistein	BSA	Self-assembly	Genistein loaded in self-assembled bovine serum albumin nanovehicles and their effects on mouse mammary adenocarcinoma cells	Ferrado et al. (2021)	2021
3	Chrysin	BSA	Self-assembly	Chrysin-loaded bovine serum albumin particles as bioactive nanosupplements	Ferrado et al. (2020)	2020
4	Chrysin	BSA	Self-assembly	Formation and characterization of self-assembled bovine serum albumin nanoparticles as chrysin delivery systems	Ferrado et al. (2019)	2019
5	Curcumin	HSA	Self-assembly	Rapid generation of homogenous tumor spheroid microtissues in a scaffold-free platform for high-throughput screening of a novel combination nanomedicine	Abolhassani et al. (2023)	2023
6	Curcumin	HSA	Emulsification	A Novel Delivery System of RGD-HSA Loaded GEM/CUR Nanoparticles for the Treatment of Pancreatic Cancer Therapy	Ma et al. (2022)	2022
7	Curcumin	BSA	Self-assembly	Self-assembled albumin nanoparticles for redox responsive release of curcumin	E et al. (2022)	2022
8	Curcumin	BSA	Self-assembly	Multifunctionalized Protein-Based Drug Delivery System for Inhibition of Tumor Growth and Progression	Qin et al. (2020)	2021
9	Curcumin	HSA	Desolvation	HSA-curcumin nanoparticles: a promising substitution for Curcumin as a Cancer chemoprevention and therapy	Matloubi and Hassan (2020)	2020
10	Curcumin	Camel serum albumin (CSA)	Self-assembly	GSH-responsive curcumin/doxorubicin encapsulated Bactrian camel serum albumin nanocomposites with synergistic effect against lung cancer cells	Yu et al. (2019)	2020
11	Curcumin	HSA	Self-assembly	Synergistic Effect of Self-Assembled Curcumin and Piperine Co-Loaded Human Serum Albumin Nanoparticles on Suppressing Cancer Cells	Abolhassani et al. (2020)	2020
12	Curcumin	HSA	Self-assembly	Albumin-based lipoprotein nanoparticles for improved delivery and anticancer activity of curcumin for cancer treatment	Kumari et al. (2020)	2020
13	Curcumin	BSA	Nab^TM^	Indocyanine Green and Curcumin Co-Loaded Nano-Fireball-Like Albumin Nanoparticles Based on Near-Infrared-Induced Hyperthermia for Tumor Ablation	Pham et al. (2020)	2020
14	Curcumin	BSA	Desolvation	Curcumin-human serum albumin nanoparticles decorated with PDL1 binding peptide for targeting PDL1-expressing breast cancer cells	Hasanpoor et al. (2020)	2020
15	Curcumin	BSA	Self-assembly	Effect of Biotin-Targeted Protein-Based Nanoparticles Contain of Curcumin on the Expression of Apoptotic Index Bax and Bcl2 Proteins	Amiri et al. (2020)	2020
16	Curcumin	HSA	Desolvation	Aptamer functionalized curcumin-loaded human serum albumin (HSA) nanoparticles for targeted delivery to HER-2 positive breast cancer cells	Saleh et al. (2019)	2019
17	Curcumin	BSA	Self-assembly	Multifunctional nanoparticles from albumin for stimuli-responsive efficient dual drug delivery	Nosrati et al. (2019)	2019
18	Curcumin	BSA	Desolvation	Evaluation of Intestinal Absorption Mechanism and Pharmacokinetics of Curcumin-Loaded Galactosylated Albumin Nanoparticles	Huang et al. (2019)	2019
19	Myricetin	BSA	Desolvation	Design, *in silico* modelling and functionality theory of folate-receptor-targeted myricetin-loaded bovine serum albumin nanoparticle formulation for cancer treatment	Kunjiappan et al. (2020)	2020
20	Resveratrol	HSA	Emulsification	RGD-Conjugated Resveratrol HSA Nanoparticles as a Novel Delivery System in Ovarian Cancer Therapy	Long et al. (2020)	2020
21	Resveratrol	BSA	Self-assembly	Drug-binding albumins forming stabilized nanoparticles for co-delivery of paclitaxel and resveratrol: *In vitro* / *in vivo* evaluation and binding properties investigation	Zhao et al. (2020)	2020
22	Resveratrol	HSA	Emulsification	Folate-conjugated human serum albumin-encapsulated resveratrol nanoparticles: preparation, characterization, bioavailability and targeting of liver tumors	Lian et al. (2019)	2019
23	Quercetin	BSA	Desolvation	Chemosensitizer and docetaxel-loaded albumin nanoparticle: overcoming drug resistance and improving therapeutic efficacy	Desale et al. (2018)	2018
24	Piceatannol	BSA	Desolvation	Albumin Nano-Encapsulation of Piceatannol Enhances Its Anticancer Potential in Colon Cancer Via Downregulation of Nuclear p65 and HIF-1α	Aljabali et al. (2020)	2020
25	Naringenin	BSA	Desolvation	Fabrication of bovine serum albumin-polyethylene glycol nanoparticle conjugated-folic acid loaded-naringenin as an efficient carrier biomacromolecule for suppression of cancer cells	Firouzabadi et al. (2023)	2023
26	Kaempferol	α-lactalbumin (ALA)	Desolvation	Enhanced cytotoxicity and antioxidant capacity of kaempferol complexed with α-lactalbumin	Diao et al. (2021)	2021
27	Myricetin	BSA	Desolvation	Design, *in silico* modelling and functionality theory of folate-receptor-targeted myricetin-loaded bovine serum albumin nanoparticle formulation for cancer treatment	Kunjiappan et al. (2020)	2020
28	Carnosic acid	BSA	Desolvation	Carnosic Acid Encapsulated in Albumin Nanoparticles Induces Apoptosis in Breast and Colorectal Cancer Cells	Khella et al. (2022)	2022
29	Thymol	BSA	Desolvation	Study the Anticancer Properties of Thymol-Loaded PEGylated Bovine Serum Albumin Nanoparticles Conjugated with Folic Acid	Al-Salih et al. (2023)	2023
30	Gallic acid	HSA	Desolvation	Preparation and *in vitro* characterization of gallic acid-loaded human serum albumin nanoparticles	Mohammad-Beigi et al. (2015)	2015
31	Gallic acid	BSA	Self-assembly	Paclitaxel-Induced Ultrasmall Gallic Acid-Fe@BSA Self-Assembly with Enhanced MRI Performance and Tumor Accumulation for Cancer Theranostics	An et al. (2018)	2018
32	Dopamine	HSA	Desolvation	Dual-targeted drug delivery system based on dopamine functionalized human serum albumin nanoparticles as a carrier for methyltestosterone drug	Noori et al. (2021)	2021

Phenolic compounds have gained attention owing to their diverse biological activities and pharmacological effects, such as antioxidant, anti-inflammatory, anticancer, and anti-viral activities. However, the clinical use of certain phenolic compounds is limited due to their instability, low solubility, short half-life, and high toxicity. Drug-loaded albumin NPs enhance the stability, solubility, and half-life of these drugs while also facilitating their targeted delivery. The drugs can be locally released, thereby reducing systemic toxicity, improving anticancer efficacy, and making them more promising for clinical use. However, compared with research on other types of nanocarriers, the anti-tumor research on phenolic compounds based on albumin NPs is lacking. In line with the good performance of phenolic compounds and the brilliant achievements of this class of drugs in the field of anti-tumor nanocarriers, including albumin NPs, the research on incorporating phenolic compounds into albumin NPs to improve their efficacy and safety or exerting a synergistic anti-tumor effect with other drugs could be a research hotspot in the future.

#### 4.2.5 Combination therapy

**FIGURE 7:**
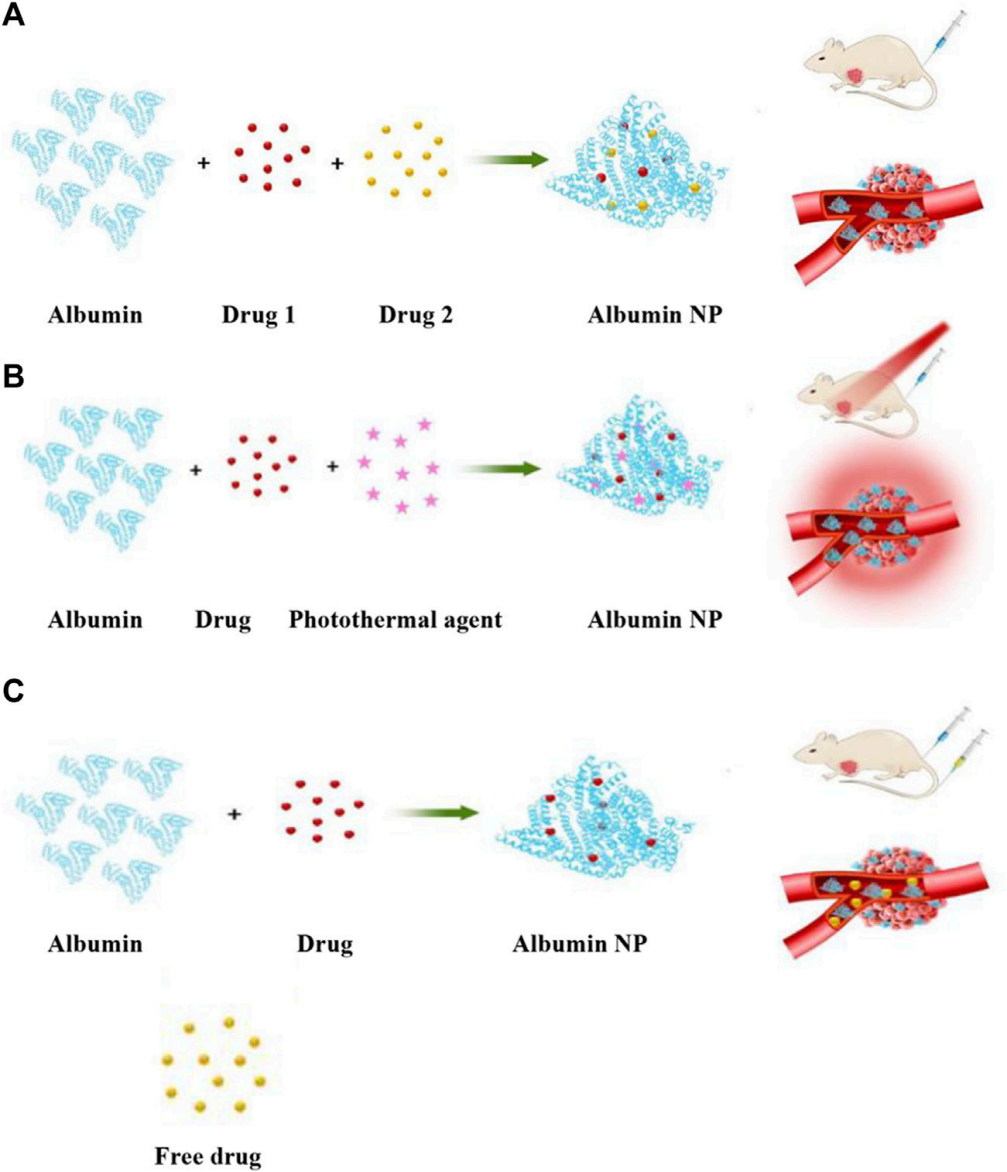
**(A)** Co-loading of different therapeutic molecules into the same albumin NP. **(B)** Co-loading of substances such as photosensitive molecules or photothermal agents with therapeutic molecules into the same albumin NP. **(C)** Combination of albumin NP drugs with another free drug.

Achieving the desired anti-tumor effect solely through a single treatment method is difficult because of the considerable heterogeneity and genetic instability of tumors, as well as their complex pathogenic mechanisms. Based on in-depth research on the interaction mechanisms among different treatment methods, combination therapy has gained considerable momentum in recent years. This approach involves combining targeted therapy, cytotoxic chemotherapy, and immunotherapy. Combination therapy can include the combination of different therapeutic drugs or modalities. Multifunctional NPs are commonly used for combination therapy. Albumin NPs are useful owing to their large specific surface area and the availability of multiple groups for chemical modification. They can be used to co-load different therapeutic molecules (Liu et al., 2018; Hao et al., 2019), such as paclitaxel and resveratrol, enabling the synergistic effect of the two drugs and overcoming resistance to paclitaxel (Zhao et al., 2020) (Figure 7A). Besides therapeutic drugs, photosensitive molecules and photothermal agents can also be loaded onto albumin NPs. These substances are typically hydrophobic and require organic solvents for dissolution, which can be toxic. Furthermore, they lack tumor-targeted accumulation and are easily cleared from the body (Li et al., 2020). These deficiencies have limited the potential for clinical translation of these substances. However, these limitations can be resolved by loading them onto albumin NPs.

Unlike free drugs, chemotherapy drugs, immune adjuvants, and photosensitizers can be loaded into the same NP through the albumin multifunctional nanoplatform and enter the tumor tissue at the same time, enabling a variety of treatments to be performed simultaneously or in sequence. This development allows for complementary relationships between various therapies that overcome each other’s deficiencies and improve the overall therapeutic effect. Therefore, albumin NP drug-loading technology has sparked the development of novel anticancer technologies, such as phototherapy (including PDT and PTT), thermotherapy, and sonodynamic therapy. Consequently, multifunctional NPs can be used to achieve combination therapy with novel anticancer technologies or conventional chemotherapy (Battogtokh and Ko, 2017; Pham et al., 2020; Chen et al., 2022; Ye et al., 2023) (Figure 7B). For instance, PDT is a non-invasive cancer treatment method that selectively treats malignant lesions through photodynamic responses. Under the irradiation of a specific wavelength of laser, a photosensitizer produces a large number of ROS at the tumor site to kill tumor cells and inhibit tumor growth. Furthermore, ROS prevents the chemical resistance caused by the scavenging of ROS by the thioredoxin system and increases the effect of chemotherapy. Xu et al. prepared multifunctional albumin NPs by co-carrying porphyrin IX and paclitaxel in albumin NPs to prevent chemoresistance and increase the effect of chemotherapy while performing PDT. Notably, these NPs improved the efficacy of chemotherapy and PDT and inhibited chemotherapy resistance (Xu et al., 2023).

In addition, the production of ROS in PDT intensifies local hypoxia in the hypoxic TME, offering the possibility of releasing hypoxia-activated prodrugs (HAPs). HAPs can be activated into highly cytotoxic substances under hypoxia conditions to selectively kill hypoxic cells in solid tumors and have low cytotoxicity to normal tissues. However, owing to the uneven distribution of hypoxic levels among tumors, the release efficiency of HAPs is usually limited by the degree of hypoxia, which leads to unsatisfactory therapeutic effects. The increased hypoxia of tumors caused by PDT can improve the efficiency of HAP release, and the combination of the two therapies can also effectively improve the treatment effect (Yuan et al., 2022; Xu et al., 2024). In addition, PDT can induce a systemic anti-tumor immune response through immunogenic cell death (ICD) to further strengthen the anti-tumor effect of PDT. However, this immune response is easily tolerated by tumor cells through a variety of molecular and cellular mechanisms, including the immune checkpoint pathway. Therefore, immune checkpoint inhibitors can simply overcome this deficiency, providing a scientific basis for the combination of immune checkpoint blockade therapy (ICBT) and PDT. Wang et al. constructed an albumin-based nanoplatform for co-delivery of IR780, NLG919 dimer, and a hypoxia-activated prodrug tirapazamine (TPZ) as the dual enhancer for synergistic cancer therapy. IR780 generates ^1^O_2_ under NIR irradiation for PDT and triggers the release of TPZ, activating its chemotherapy via exacerbated tumor hypoxia. The study also found that TPZ-mediated chemotherapy boosts PDT-induced tumor ICD, inducing stronger anti-tumor immunity. In addition, the enriched intratumoral glutathione triggered the activation of NLG919 to mitigate the immunosuppressive TME by specifically inhibiting indoleamine 2 and 3-dioxygenase 1 (IDO-1). This promoted the intratumoral infiltration of cytotoxic T lymphocytes and the suppression of primary and distant tumors, whereas the generated memory T cells suppressed tumor recurrence and metastasis by approximately 100%. In summary, the developed albumin multifunctional nanoplatform enabled dual enhanced photodynamic immunotherapy for breast cancer via hypoxia-activated chemotherapy (Wang et al., 2023).

Similarly, combination therapy may involve combining drug-loaded albumin NPs with another free drug (Chen et al., 2022; Schaal et al., 2022) (Figure 7C). The most commonly used albumin nanodrug in this combination modality is nab-paclitaxel, as it is the first and currently most widely used albumin nanodrug in oncology. This combination therapy is still a research hotspot, involving the study of the efficacy and safety of platinum, fluorouracil, immune checkpoint inhibitors, and other antineoplastic drugs combined with nap-paclitaxel in the treatment of various refractory tumors to provide a basis for clinical medication (Paz-Ares et al., 2018; Yin et al., 2020; Kase et al., 2023). Jiang et al. used torpalimab plus nab-paclitaxel for metastatic or recurrent TNBC in a randomized phase Ⅲ trial and found that this combination improved progression-free survival with an acceptable safety profile for PD-L1-positive patients with metastatic or recurrent TNBC (Jiang et al., 2024).

In summary, combination therapy is a super-additive approach, facilitating the mutual promotion and complementation of multiple treatment methods or drugs, resulting in greater efficacy than each individual treatment method/drug alone. This approach also helps prevent multidrug resistance, improves the body’s tolerance, and ultimately contributes to an improvement in therapeutic indices (Chou, 2010; Hatami et al., 2022).

#### 4.2.6 Clinical applications

Albumin has been widely used as a carrier for drug delivery in oncological research. Abraxane^®^ is a prime example of clinical success among all FDA-approved cancer nanomedicines (Esim and Hascicek, 2021; Meng et al., 2023). On 23 November 2021, the FDA approved Fyarro^®^, the second product developed and marketed using albumin-based technology, to treat adults with locally advanced unresectable or metastatic malignant PEComa. Furthermore, various chemotherapy drugs that use albumin NPs, such as albumin-bound docetaxel, SYHX 2011 (modified albumin-bound paclitaxel), and AR60 (Abraxane/Rituximab 160 nm NP), are currently undergoing clinical trials and have not yet been released on the market (Table 12). Notably, only two albumin-bound anti-tumor drugs have been launched since the introduction of Abraxane^®^ in 2005 (generic versions of Abraxane^®^ from different countries are not included). Most research on the use of albumin NPs in oncology is limited to animal experiments, with a low rate of clinical translation. Moreover, although albumin-based nanomedicines have shown greater efficacy than free drugs, they still encounter the same obstacles as traditional drugs, such as drug resistance and inadequate drug accumulation in tumors. Notably, owing to the EPR heterogeneity, albumin-based nanomedicines exhibit differences in treatment outcomes across various tumors, patients, or even different times in the same patient (Kunde and Wairkar, 2022a; Kong et al., 2023). Therefore, albumin–drug loading technology is still underdeveloped and requires significant investment in scientific research for further improvement.

**TABLE 12:** New anti-tumor drugs based on albumin nanotechnology in clinical trials.

No.	Trial identifier no.	Phase	Status	Conditions	Drug/Therapeutic agent
1	NCT04471675	Ⅰ	Recruiting	Advanced Solid Tumors	Albumin-bound docetaxel
2	NCT05705635	Ⅱ	Recruiting	Gastric Adenocarcinoma; Adenocarcinoma of Gastroesophageal Junction	Albumin-bound docetaxel; Taxotere
3	NCT05027204	Ⅰ/Ⅱ	Recruiting	Squamous Cell Carcinoma of Head and Neck	Albumin-bound docetaxel; Nivolumab
4	NCT05616494	Ⅱ	Recruiting	Pancreatic Cancer	Albumin-bound docetaxel
5	NCT05325229	Ⅱ	Recruiting	Platinum-resistant Recurrent Ovarian Cancer	Albumin-bound docetaxel; Bevacizumab
6	NCT05114915	Ⅰ	Unknown status	Advanced Solid Tumors	Albumin-bound docetaxel
7	NCT06136988	Ⅰ/Ⅱ	Not yet recruiting	Locally Advanced Unresectable Esophageal Squamous Carcinoma	Albumin-bound docetaxel; SG001; Cisplatin; Paclitaxel
8	NCT05753865	Ⅲ	Not yet recruiting	Advanced Breast Cancer	SYHX 2011; Albumin-bound paclitaxel
9	NCT03003546	Ⅰ	Completed	Recurrent Aggressive Non-Hodgkin Lymphoma; Recurrent B-Cell Non-Hodgkin Lymphoma; Recurrent Small Lymphocytic Lymphoma; Refractory Aggressive Non-Hodgkin Lymphoma; Refractory B-Cell Non-Hodgkin Lymphoma; Refractory Small Lymphocytic Lymphoma	Nab-paclitaxel/Rituximab-coated Nanoparticle AR160
10	NCT05125523	Ⅰ	Recruiting	Advanced Solid Tumors	Albumin-bound sirolimus

In current clinical research on albumin NPs for anti-tumor purposes, breast, pancreatic, lung, and gastric cancers are the most widely investigated tumors (Table 9; Figure 3B). Breast cancer is a highly malignant tumor that poses a serious threat to women’s health. Notably, the number of patients with breast cancer worldwide is predicted to reach 3.04 million by 2040. Breast cancer is a highly heterogeneous disease, with different phenotypes showing varying sensitivities to drug treatment, resulting in different prognoses in patients in the same stage receiving the same treatment regimen. Genetic and molecular subtyping of breast cancer is closely associated with the prognosis. Therefore, new treatment options are needed to implement precision medicine and maximize treatment benefits for the various molecular subtypes (Alamdari et al., 2022; Kunde and Wairkar, 2022b). The ongoing clinical trials of albumin NPs for breast cancer in this field are listed in Table 13. The table indicates that current breast cancer clinical trials are focused on the therapeutic effects of nab-paclitaxel combined with new drugs with different mechanisms for treating breast cancer with different molecular types, such as the tyrosine kinase inhibitor pyrotinib and the anti-PD-L1 monoclonal antibody SHR-1316.

**TABLE 13:** Ongoing clinical trials of albumin NPs in breast cancer.

No.	Trial identifier no.	Phase	Status	Conditions	Drug/Therapeutic agent
1	NCT04917900	Ⅱ	Recruiting	Her2-positive Early or Locally Advanced Breast Cancer	Pyrotinib; Albumin-bound paclitaxel; Trastuzumab
2	NCT05659056	Ⅱ	Recruiting	HER2-enriched Early or Locally Advanced Breast Cancer	Pyrotinib; Trastuzumab; Paclitaxel-albumin
3	NCT06000917	Ⅱ	Recruiting	ER+/HER2+ Early or Locally Advanced Breast Cancer	Pyrotinib; Trastuzumab; Carboplatin; Albumin paclitaxel
4	NCT05192798	Ⅱ	Recruiting	Relapsed or Metastatic Triple-negative Breast Cancer	Albumin-bound paclitaxel; Apatinib mesylate; Bevacizumab
5	NCT04734262	Ⅱ	Recruiting	Locally Recurrent or Metastatic Triple-negative Breast Cancer	Sitravatinib; Tislelizumab; Nab-paclitaxel
6	NCT05205200	Ⅱ	Recruiting	HR+/HER2- Advanced Breast Cancer	SHR-1316; SHR6390; Nab-paclitaxe; SERD; AI
7	NCT04136782	Ⅳ	Recruiting	Triple-negative Breast Cancer	Albumin-bound paclitaxel; Carboplatin; Epirubicin; Docetaxel
8	NCT05429294	Ⅱ	Recruiting	HER2-positive Advanced or Metastatic Breast Cancer	Pyrotinib; Trastuzumab; Albumin paclitaxel
9	NCT05353361	Ⅱ	Recruiting	HER2-positive breast cancer	Oleclumab SHR-A1811; Pyrotinib; Pertuzumab; Adebrelimab; Albumin-bound paclitaxel
10	NCT04137653	Ⅲ	Recruiting	Triple-negative Breast Cancer	Nab-Paclitaxel; Carboplatin; Paclitaxel
11	NCT05390710	Ⅰ/Ⅱ	Recruiting	Locally Advanced or Metastatic Triple-negative Breast Cancer	LAE005; Nab-paclitaxel; Afuresertib
12	NCT05227664	Ⅱ	Recruiting	Metastatic Triple-negative Breast Cancer	AK117; AK112; Nab-paclitaxel; Paclitaxel
13	NCT05068141	Ⅱ	Recruiting	Advanced Triple-negative Breast Cancer	SG001; Nab-paclitaxel
14	NCT04958785	Ⅱ	Recruiting	Locally Advanced or Metastatic Triple-negative Breast Cancer	Magrolimab; Nab-paclitaxel; Paclitaxel; Sacituzumab; Govitecan-hziy

Pancreatic cancer is a highly aggressive malignant disease commonly referred to as the “king of cancer” owing to its rapid progression, poor treatment response, and short survival. The most common type of pancreatic cancer is pancreatic ductal adenocarcinoma (PDAC), accounting for 90% of all cases. It is predicted to be the second leading cause of cancer death in the next 20 years (Elsayed and Abdelrahim, 2021; Sung et al., 2021). Pancreatic cancer has a high KRAS mutation rate, with nearly 90% of cases being RAS-dependent. Research suggests that this mutation is a major driver of PDAC onset and progression (Spaargaren et al., 1995; Scheffzek et al., 1997). Furthermore, albumin and albumin-related drugs accumulate in KRAS-mutated pancreatic cancer cells and tissues. Therefore, nano-delivery systems based on serum albumin are widely used in pancreatic cancer treatment (Kratz, 2008). Abraxane^®^ is currently the only albumin-bound drug approved to treat metastatic pancreatic cancer. However, new albumin-based nanomedicines for pancreatic cancer are also in development. Table 14 lists ongoing clinical trials of albumin NPs for pancreatic cancer in this field, which currently focus on the development of new drugs such as albumin-bound docetaxel and combination treatment regimens based on nab-paclitaxel.

**TABLE 14:** Ongoing clinical trials of albumin NPs in pancreatic cancer.

No.	Trial identifier no.	Phase	Status	Conditions	Drug/Therapeutic agent
1	NCT05346146	Ⅱ	Recruiting	UnresecTable Locally Advanced Pancreatic Cancerr	Sintilimab; Gemcitabine; Albumin-paclitaxel
2	NCT05035147	Ⅳ	Recruiting	Locally Advanced or Metastatic PancreaticCancer	Albumin-bound paclitaxel; Gemcitabine
3	NCT05168527	Ⅱ	Recruiting	Liver Metastases From Pancreatic Cancer	Fruquintinib, Albumin-paclitaxel, Gemcitabine
4	NCT05616494	Ⅱ	Recruiting	Pancreatic Cancer	Albumin-bound docetaxel
5	NCT05827796	Ⅰ/Ⅱ	Recruiting	Advanced Pancreatic Cancer	IN10018; Albumin-bound paclitaxel; Gemcitabine; KN046
6	NCT04524702	Ⅱ	Recruiting	Advanced or Metastatic Pancreatic Cancer	Gemcitabine; Hydroxychloroquine; Nab-paclitaxel; Paricalcitol
7	NCT04617821	Ⅲ	Recruiting	Borderline ReseacTable and Locally Advanced Pancreatic Cancer	Nab-paclitaxel; Gemcitabine; mFOLFIRINOX
8	NCT04158635	Ⅰ	Recruiting	UnresecTable Pancreatic Cancer	Bosentan; Gemcitabine; Nab-paclitaxel
9	NCT02340117	Ⅱ	Recruiting	Metastatic Pancreatic Cancer	SGT-53; Nab-paclitaxel; Gemcitabine
10	NCT05908747	Ⅱ	Recruiting	Locally Advanced or Borderline ResecTable Pancreatic Cancer	Surufatinib; Gemcitabine; Nab-paclitaxel
11	NCT04589234	Ⅱ	Recruiting	Metastatic pancreatic cancer	Salmonella-IL2; FOLFIRINOX; Gemcitabine; Abraxane^®^
12	NCT05497778	Ⅰ	Recruiting	Advanced Pancreatic Cancer	Gemcitabine; Nab-paclitaxel; IM156
13	NCT05371223	Ⅱ	Recruiting	Pancreatic Cancer Peritoneal Metastases	Nab-paclitaxel; Gemcitabine
14	NCT04151277	Ⅱ	Recruiting	Metastatic Pancreatic Cancer	FOLFOX-A; Gemcitabe; Abraxane^®^; G-CSF
15	NCT04940286	Ⅱ	Recruiting	ResecTable/Borderline ResecTable Primary Pancreatic	Durvalumab; Gemcitabine; Nab-paclitaxel; Oleclumab
16	NCT03777462	Ⅱ	Recruiting	Borderline ResecTable Pancreatic Cancer	Neoadjuvant gemcitabine; Nab-paclitaxel; S-1
17	NCT05557851	Ⅰ	Recruiting	Metastatic Adenocarcinoma of the Pancreas	Minnelide; Abraxane^®^; Gemcitabine
18	NCT04570943	Ⅱ	Recruiting	Locally Advanced Pancreatic Adenocarcinoma	Gemcitabine; Nab-paclitaxel; FOLFIRINOX
19	NCT05065801	Ⅱ	Recruiting	The first metastatic line pancreatic cancer	Gemcitabine; Nab-paclitaxel; FOLFIRINOX
20	NCT05218889	Ⅰ/Ⅱ	Recruiting	Advanced Metastatic Pancreatic Cancer	Surufatinib; Camrelizumab; Nab-paclitaxel; S-1; Gemcitabinex
21	NCT05685602	Ⅰ	Recruiting	Metastatic or UnresecTable Pancreatic Cancer	Emavusertib; Gemcitabine; Nab-paclitaxel
22	NCT04672005	Ⅱ	Recruiting	Metastatic pancreatic ductal adenocarcinoma	FOLFIRINOX; Gemcitabine; Nab-paclitaxel
23	NCT04731467	Ⅰ/Ⅱ	Recruiting	Advanced solid tumors and advanced metastatic pancreatic cancer	CM-24; Nivolumab; Nab-paclitaxel; Gemcitabine; Nal-IRI/5-FU/LV
24	NCT03535727	Ⅰ/Ⅱ	Recruiting	Metastatic pancreatic cancer	Gemcitabine; Nab-paclitaxel; Capecitabine; Cisplatin; Irinotecan
25	NCT04233866	Ⅱ	Recruiting	Metastatic pancreatic cancer	Gemcitabine; Nab-Paclitaxel; 5-Fluorouracil; Leucovorin; Liposomal irinotecan
26	NCT02207465	Ⅰ	Recruiting	UnresecTable and Borderline ResecTable Pancreatic Cancer	Abraxane^®^
27	NCT05026905	Ⅱ	Recruiting	Metastatic Pancreatic Cancer	Gemcitabine; Nab-paclitaxel; S1; Oxaliplatin
28	NCT05908747	Ⅱ	Recruiting	Pancreatic Cancer	Surufatinib; gemcitabine; nab-paclitaxel

Lung cancer is one of the most common cancers and the leading cause of death in males in >90 countries worldwide. Although multiple treatment options are available, satisfactory results in lung cancer treatment are difficult to achieve because of high proliferation rate, easy early metastasis, heterogeneity, and poor prognosis. Abraxane^®^ was approved as a first-line treatment for non-small cell lung cancer in 2012. However, the use of nab-paclitaxel alone in the treatment of tumors has failed to elicit the desired outcomes (Ghosh et al., 2021). Therefore, the combination therapy based on nab-paclitaxel has been widely explored. Notably, clinical studies have focused on this combination therapy for various refractory or drug-resistant lung cancers to provide a basis for clinical drug use. Table 15 lists ongoing clinical trials of albumin NPs for lung cancer in this field.

**TABLE 15:** Ongoing clinical trials of albumin NPs in lung cancer and gastric cancer.

No.	Trial identifier No.	Phase	Status	Conditions	Drug/Therapeutic agent
1	NCT04524299	Ⅱ	Recruiting	Non-small Cell Lung Cancer	Nedaplatin; Nab-paclitaxel
2	NCT05738317	Ⅱ	Recruiting	Non-small Cell Lung Cancer	Adebrelimab; Bevacizumab; Nab-paclitaxel
3	NCT05689671	Ⅳ	Recruiting	Metastatic Non-Small Cell Lung Cancer	Atezolizumab; Nab-paclitaxel; Carboplatin
4	NCT04015778	Ⅱ	Recruiting	Non-Small Cell Lung Cancer	Nivolumab; Carboplatin; Nab-paclitaxel
5	NCT03801668	Ⅲ	Recruiting	Advanced or Recurrent Gastric Adenocarcinoma	Nab-paclitaxel; Oxaliplatin; S-1
6	NCT05705635	Ⅱ	Recruiting	Gastric Adenocarcinoma; Adenocarcinoma of Gastroesophageal Junction	Albumin-bound docetaxel; Taxotere
7	NCT04135781	Ⅲ	Recruiting	Stomach Cancer	Nab-paclitaxel; Tegafur; Oxaliplatin
8	NCT05002686	Ⅱ/Ⅲ	Recruiting	Gastric Cancer	Sintilimab; Albumin-Paclitaxel; Capecitabine
9	NCT05052931	Ⅱ	Recruiting	Gastric Cancer	Nab-paclitaxel; Oxaliplatin; S-1
10	NCT06169410	Ⅳ	Recruiting	Advanced Gastric Cancer	Nab-paclitaxel; Lobaplatin; S-1; Ramucirumab
11	NCT06046963	Ⅱ	Recruiting	Gastric Cancer	Sintilimab; S-1; Oxaliplatin; Nab-paclitaxel
12	NCT05075993	Ⅰ	Recruiting	Metastatic Esophageal; Gastric Cancer; Metastatic Head and Neck Carcinoma; Metastatic Hepatocellular Carcinoma; Metastatic HPV Related Solid Tumors; Metastatic Ovarian Carcinoma; Metastatic Soft Tissue Sarcoma; Metastatic Uveal Melanoma	LVGN3616; LVGN6051; LVGN7409; Nab-Paclitaxel; Bevacizumab; Cyclophosphamide
13	NCT06102772	Ⅱ	Recruiting	Gastric Cancer	Adebrelimab; Fruquintinib; Nab-Paclitaxel; Paclitaxel

Gastric cancer is a leading cause of cancer-related death worldwide, particularly in East Asia. Although many combination treatment regimens are available, the prognosis for patients with advanced gastric cancer remains unsatisfactory. Nab-paclitaxel is currently used to treat advanced gastric cancer, but it is reserved for second-line treatment. Furthermore, its use has only been approved in a few countries owing to the limited number of studies conducted (He et al., 2018). Moreover, the CSPC Pharmaceutical Group has developed a new drug called docetaxel for injection (albumin-bound), which was designated as an orphan drug by the FDA in 2022. This drug is indicated for gastric cancer, including gastroesophageal junction cancer. However, clinical trials for advanced gastric cancer in this field are still ongoing (Table 15). Current clinical research is primarily focused on developing new drugs, such as albumin-bound docetaxel, and studying the efficacy and safety of nab-paclitaxel-based monotherapy or combination therapy as a first- or second-line treatment for advanced gastric cancer. This research may provide more evidence for the clinical use of these treatments (Bando et al., 2018; Ju et al., 2019).

## 5 Limitations

The study has some limitations. First, the data was only retrieved from a single database, which may have resulted in the exclusion of articles published in other sources such as PubMed and Scopus. Second, our analysis only included articles published in English, which may have led to the exclusion of some articles. Third, new papers published after the search date were not included in the study because the database was kept open (Rousseau and Rousseau, 2021). Finally, although we have defined as many search terms as possible, some articles may have been missed.

## 6 Conclusion

In this study, we used bibliometric analysis to obtain an overview of the research on albumin NPs in the field of oncology. We demonstrated the trends in publications in this field since 2000, identified the most influential countries, institutions, authors, journals, and citations, and discussed the current research hotspots and frontiers in this field, including “rapid and convenient synthesis methods predominated by self-assembly,” “surface modification,” “construction of multifunctional NPs for theranostics,” “research on natural active ingredients mainly based on phenolic compounds,” “combination therapy,” and “clinical applications.” Furthermore, we discussed the challenges in the clinical application of albumin NPs in the field of oncology. However, we believe that significant continuous research efforts and investigations will facilitate the promising application of albumin NP drug-loading technology in the field of oncology in the future.

## Data Availability

The original contributions presented in the study are included in the article/Supplementary Material, further inquiries can be directed to the corresponding authors.

## Abbreviations

ALA, α-Lactalbumin; BSA, Bovine serum albumin; BLM, Bleomycin; CSA, Camel serum albumin; Ce6, Chlorine e6; EPR, Enhanced permeability and retention; FDA, Food and Drug Administration; FA, Folic acid; GBCA, Gadolinium-based contrast agents; HSA, Human serum albumin; HA, Hyaluronic acid; HAPs, Hypoxia activated prodrugs; ICG, Indocyanine green; ICD, Immunogenic cell death; ICBT, Immune checkpoint blockade therapy; IDO-1, Indoleamine 2, 3-dioxygenase 1; MRI, Magnetic resonance imaging; NIRFI, Near-infrared fluorescence imaging; NPs, Nanoparticles; PDAC, Pancreatic ductal adenocarcinoma; PDT, Photodynamic therapy; PEComa, Perivascular epithelioid cell tumor; PPy, Polypyrrole; PTT, Photothermal therapy; ROS, Reactive oxygen species; SPARC, Secreted protein acidic and rich in cysteine; TNBC, Triple-negative breast cancer; TME, Tumor microenvironment; WOSCC, Web of Science Core Collection.
