# Supplementary material for: Trend of albumin nanoparticles in oncology: a bibliometric analysis of research progress and prospects
Source: Front Pharmacol. 2024 Jul 12;15:1409163. doi: 10.3389/fphar.2024.1409163 (PMC11272567; doi:10.3389/fphar.2024.1409163)
Supplement: Supplementary file 1 [file Image1.pdf]

Supplementary material

Supplementary Figures

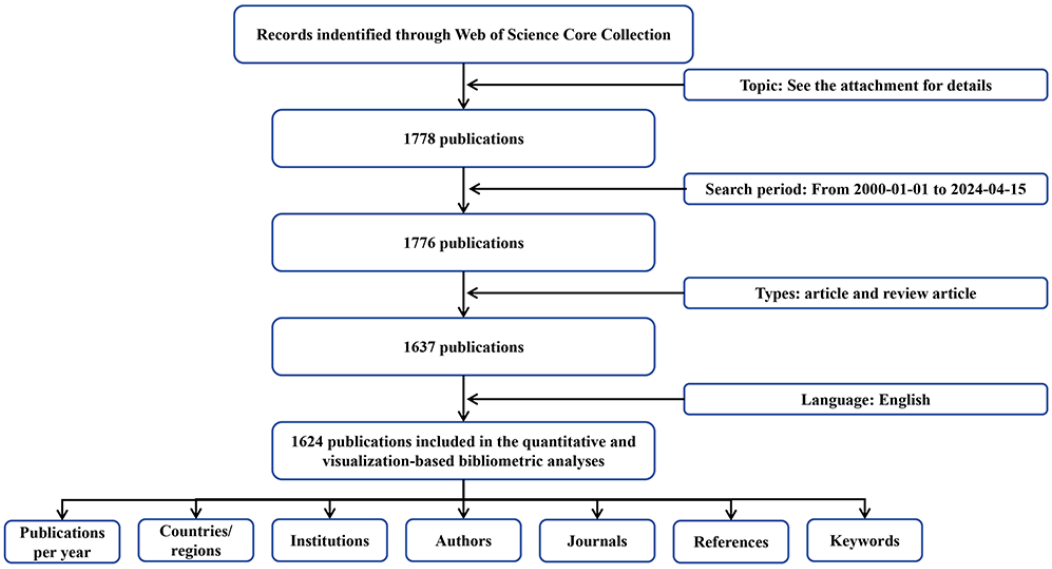

Supplementary Figure 1. Flowchart for the research's search process.

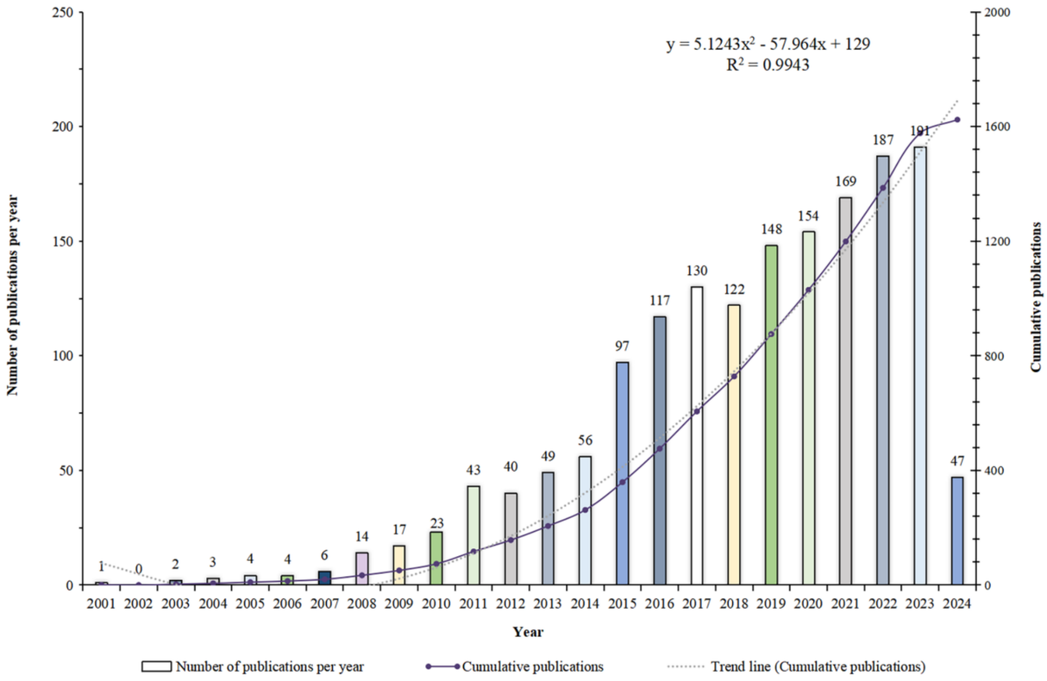

Supplementary Figure 2. Trends in the number of publications on albumin NPs in cancer.

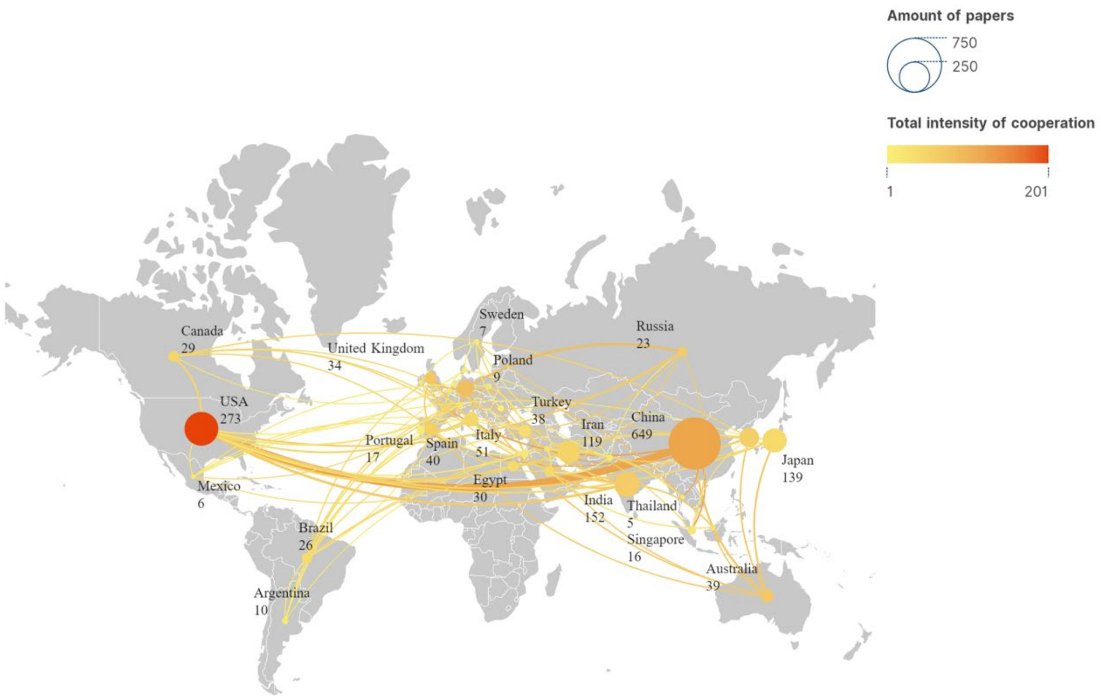

Supplementary Figure 3. VOSviewer network visualization map of countries. The minimum number of publications per country was set at five, and countries or regions that met this condition were included in the map. The color gradient indicates the total intensity of cooperation between a given country and other countries. The thickness of the line connecting the circles indicates the intensity of cooperation between the countries. The size of the circles is positively correlated with the number of articles published by a given country.

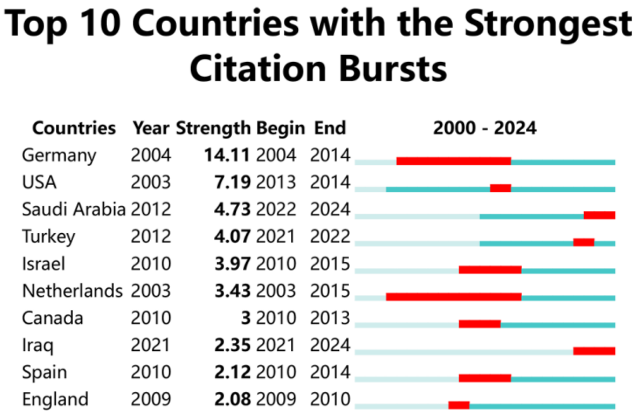

Supplementary Figure 4. Top 10 Countries with the Strongest Citation Bursts from January 1, 2000, to April 15, 2024**.** The country-citation burst map shows the sudden surge in the number of publications for a given country within a specific period. The red areas in the figure represent the period of citation bursts for each country.

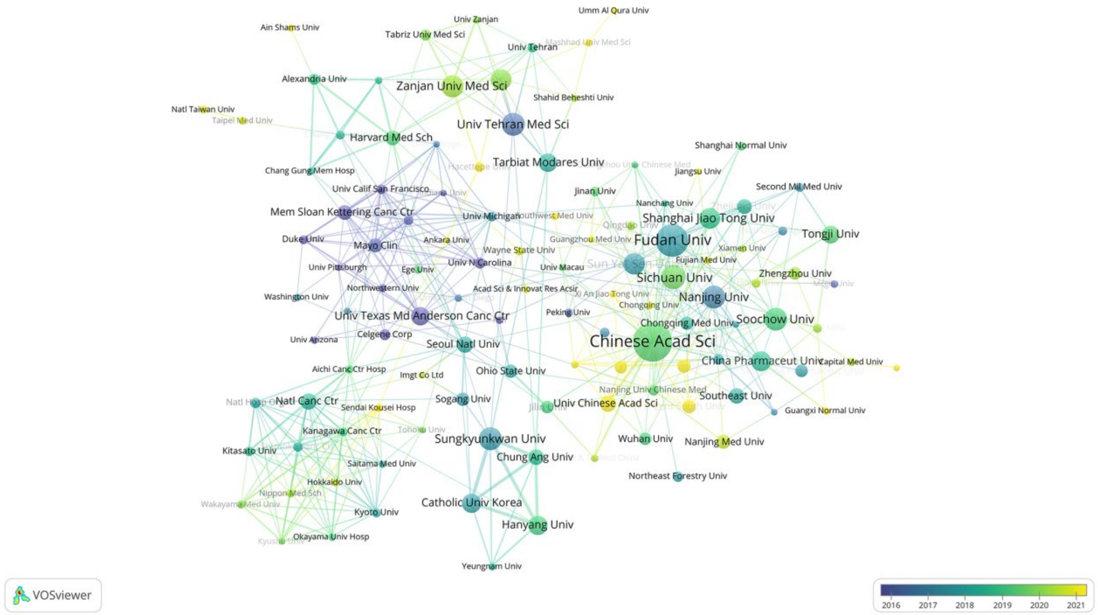

Supplementary Figure 5. VOSviewer network visualization map of research institutions. The minimum number of publications per institution was set at five. Each circle and label form one node. The size of the circle is positively correlated with the number of articles published by an institution. The color of each circle indicates the average year of appearance according to the color gradient in the lower right corner. Blue represents institutions that have been published earlier and yellow represents institutions that have published more recently. The thickness of the line connecting the circles indicates the intensity of cooperation between institutions.

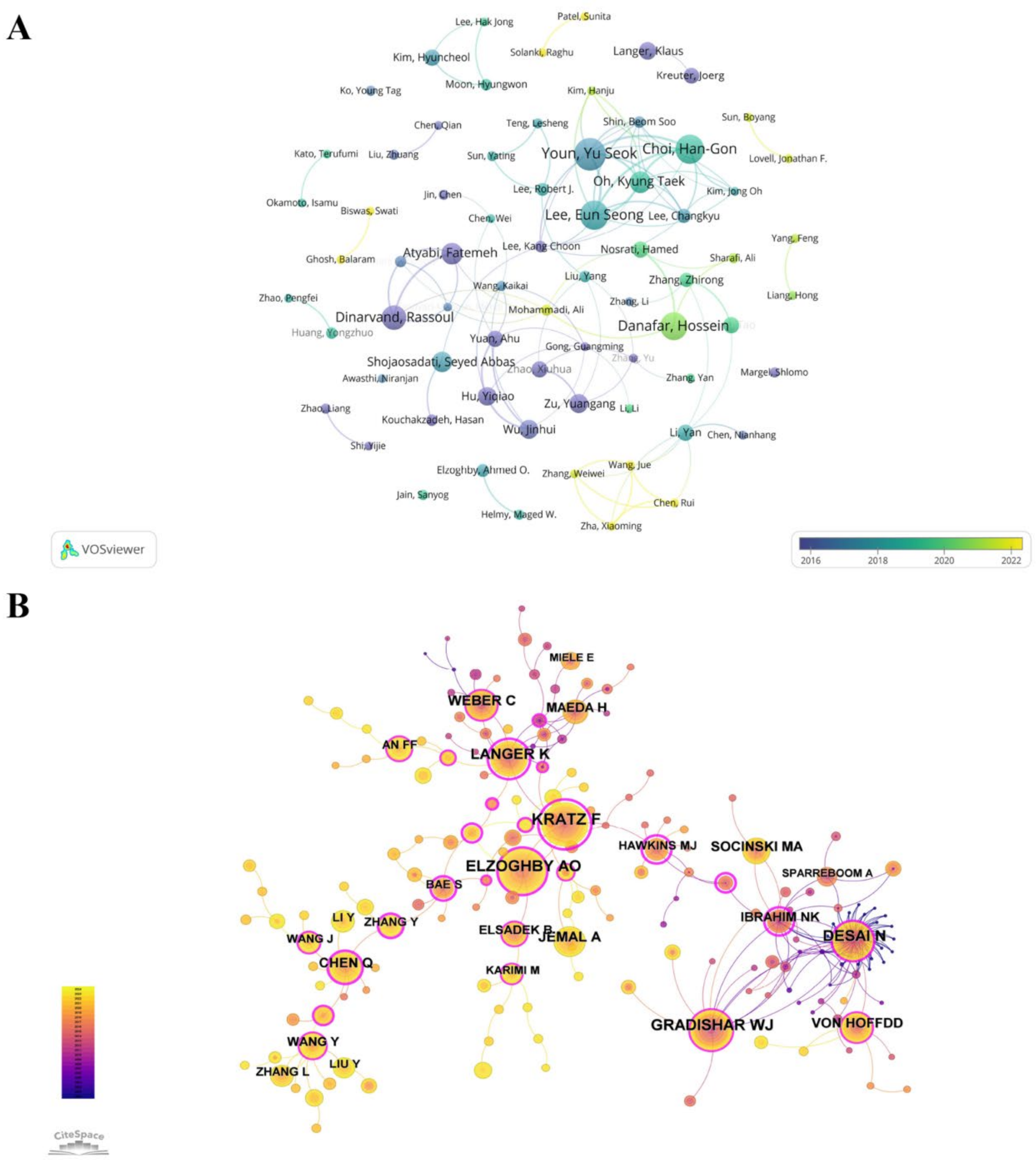

Supplementary Figure 6. The cooperation map of the authors (**A**) and the co-citation map of the authors (**B**) visualized using VOSviewer. The size of the circle in Fig A is positively correlated with the number of articles published by the author. The lines connecting the circle represent the co-occurrence of the authors, which suggests a cooperative relationship between the authors. The circle size in Fig B is positively correlated with the number of author co-citations. The lines connecting the circle represent co-cited authors. Different colors indicate different times of publication, and overlapping colors indicate that the author had published articles in all corresponding years.

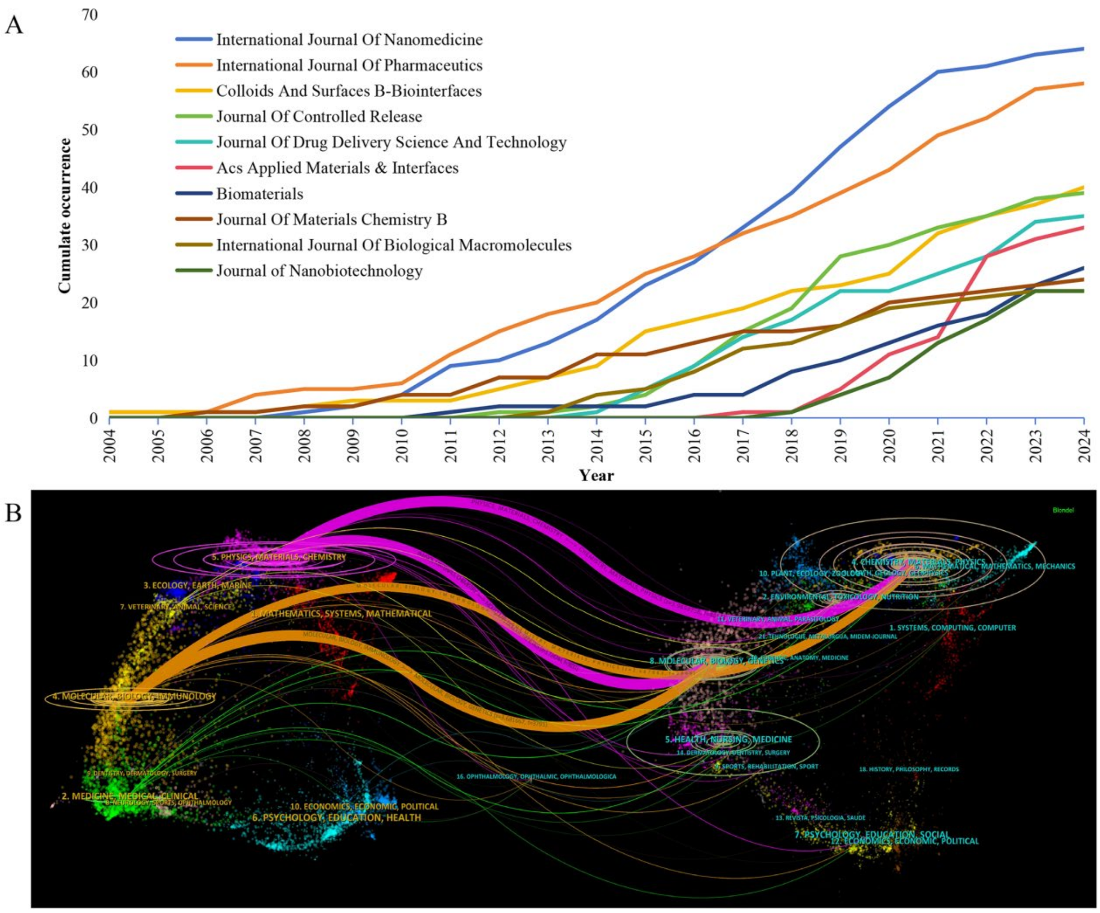

Supplementary Figure 7. (A) The changing trend of annual publication quantity in the top 10 journals. **(B)** Dual-map overlap of journals on albumin NPs for cancer research.
